# Therapeutic effects and mechanisms of plant-derived natural compounds against intestinal mucositis

**DOI:** 10.3389/fphar.2022.969550

**Published:** 2022-09-21

**Authors:** Cailan Li, Jianhui Xie, Jiahao Wang, Ying Cao, Min Pu, Qihai Gong, Qiang Lu

**Affiliations:** ^1^ Department of Pharmacology, Zunyi Medical University, Zhuhai Campus, Zhuhai, China; ^2^ Key Laboratory of Basic Pharmacology of Ministry of Education and Joint International Research Laboratory of Ethnomedicine of Ministry of Education, Zunyi Medical University, Zunyi, China; ^3^ Key Laboratory of Basic Pharmacology of Guizhou Province and School of Pharmacy, Zunyi Medical University, Zunyi, China; ^4^ The Second Affiliated Hospital of Guangzhou University of Chinese Medicine, Guangzhou, China; ^5^ Department of Pharmaceutical Sciences, Zunyi Medical University, Zhuhai Campus, Zhuhai, China

**Keywords:** intestinal mucositis, natural products, therapeutic action, mechanism, plants

## Abstract

Intestinal mucositis is a clinically related adverse reaction of antitumor treatment. Majority of patients receiving high-dose chemical therapy, radiotherapy, and bone-marrow transplant suffer from intestinal mucositis. Clinical manifestations of intestinal mucositis mainly include pain, body-weight reduction, inflammatory symptom, diarrhea, hemoproctia, and infection, which all affect regular nutritional input and enteric function. Intestinal mucositis often influences adherence to antitumor treatment because it frequently restricts the sufferer’s capacity to tolerate treatment, thus resulting in schedule delay, interruption, or premature suspension. In certain circumstances, partial and general secondary infections are found, increasing the expenditures on medical care and hospitalization. Current methods of treating intestinal mucositis are provided, which do not always counteract this disorder. Against this background, novel therapeutical measures are extremely required to prevent and treat intestinal mucositis. Plant-derived natural compounds have lately become potential candidates against enteric injury ascribed to the capacity to facilitate mucosal healing and anti-inflammatory effects. These roles are associated with the improvement of intestinal mucosal barrier, suppression of inflammatory response and oxidant stress, and modulation of gut microflora and immune system. The present article aims at systematically discussing the recent progress of plant-derived natural compounds as promising treatments for intestinal mucositis.

## Introduction

Intestinal mucositis, a clinically significant adverse reaction of antitumor treatment, is characterized by ulcerative lesions along the gastroenteric mucosa (Dahlgren et al., 2021). It appears in nearly 40% of sufferers adopting standard-dose chemotherapy, and over 60% of sufferers receiving high-dose chemotherapy, radiotherapy, and bone-marrow transplantation (Sougiannis et al., 2021). Intestinal mucositis is not only related to a series of remarkable adverse symptoms, including serious diarrhea in 5%–44% of sufferers, obvious body-weight reduction, and decreased nutrient uptake but also associated with the restricted ability of sufferers to tolerate therapy, thus resulting in the delay of succeeding cycles or premature drug withdrawal (Miknevicius et al., 2021).

The clinical signs of intestinal mucositis result from epithelial damage, which is followed by a range of complicated biological events that occur in the diverse cellular and tissular chambers of the mucosa (Batista et al., 2020). Localized and general secondary infections, which increase the expenditures of medical treatment and hospitalization, are found under certain circumstances (Tooley et al., 2009). Therefore, intestinal mucositis poses a significant negative effect on sufferer’s clinical outcome, and its complications can even cause death in serious sufferers (van Vliet et al., 2010). There are still no prevention strategies or appropriate therapies for intestinal mucositis. Current treatments primarily focus on relieving the symptoms of intestinal mucositis. To assist the clinical governance of intestinal mucositis, the MASCC/ISOO formulated a guideline including updated information on alternative treatments for this disease (Prisciandaro et al., 2011). These consist of mucosal coating agents, opiates and painkillers, growth factors and cytokines, antimicrobial agents, cryotherapy, and natural drugs (Kissow, 2015).

**FIGURE 1:**
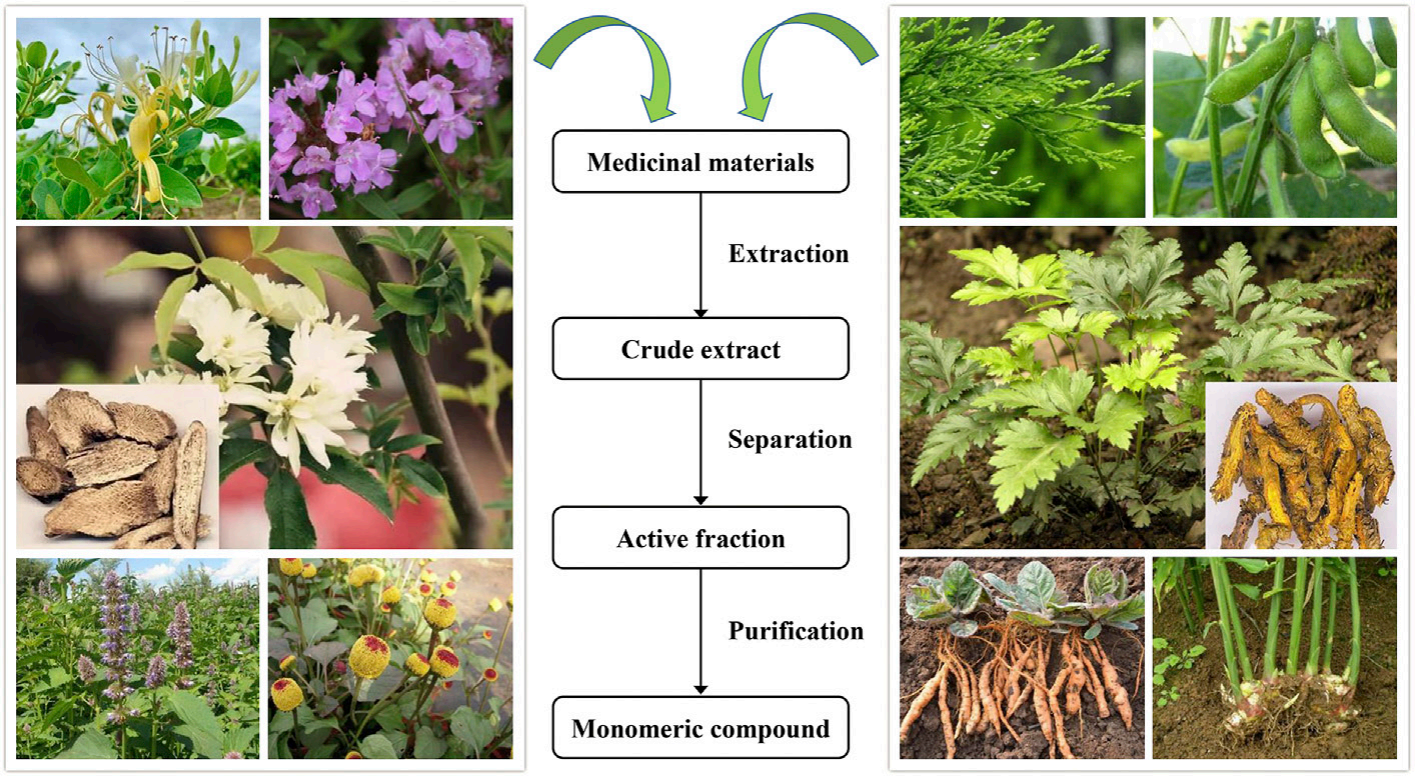
Various plants of natural compounds for treating intestinal mucositis in the past few decades.

Under the circumstances, natural products have attracted much attention and recognition as their prominent bioactivities, including anti-inflammation, antioxidation, immune regulation, and metabolic functions, in the prevention and treatment of intestinal mucositis, cancers, angiocardiopathies, and metabolic and respiratory disorders (Cheah et al., 2014a; da Silva et al., 2021; Wright et al., 2009). A range of preclinical research works have proven that various natural compounds from plants (Figure 1) exerted preventive and therapeutical actions on intestinal mucositis through diversified mechanisms including relieving oxidative injury, decreasing inflammatory reaction, maintaining gut barrier function, and modulating the structure of intestinal microbiome, manifesting that natural compounds possess the potential to stop the progress of intestinal mucositis and delay the clinical course of this disorder (Ribeiro et al., 2016). Thus, in this article, we generalize the pharmacological mechanisms of natural compounds, such as berberine, quercetin, curcumin, baicalein, and luteolin, in preventing and treating intestinal mucositis. These were envisaged to contribute to offering novel ideas for the development of new drugs against intestinal mucositis.

## Methods

For recognizing the studies associated with the effect and mechanism of natural compounds against intestinal mucositis, our team consulted the articles in the following scientific databases from inception until July 2022: Web of Science, PubMed, Google Scholar, and CNKI. The keyword “intestinal mucositis” was adopted for the literature retrieval. All articles with abstracts were taken into account.

After retrieval, the gained articles were strictly filtered. The first screening was conducted in line with the titles and abstracts. The second screening was performed on the basis of the full-text. Finally, all the studies fitting the theme were gathered as the supportive resource of the present review. The exclusion criteria were as follows: crude extracts or oils, not plant-derived natural compounds, and combined therapies.

## Natural compounds against intestinal mucositis

**FIGURE 2:**
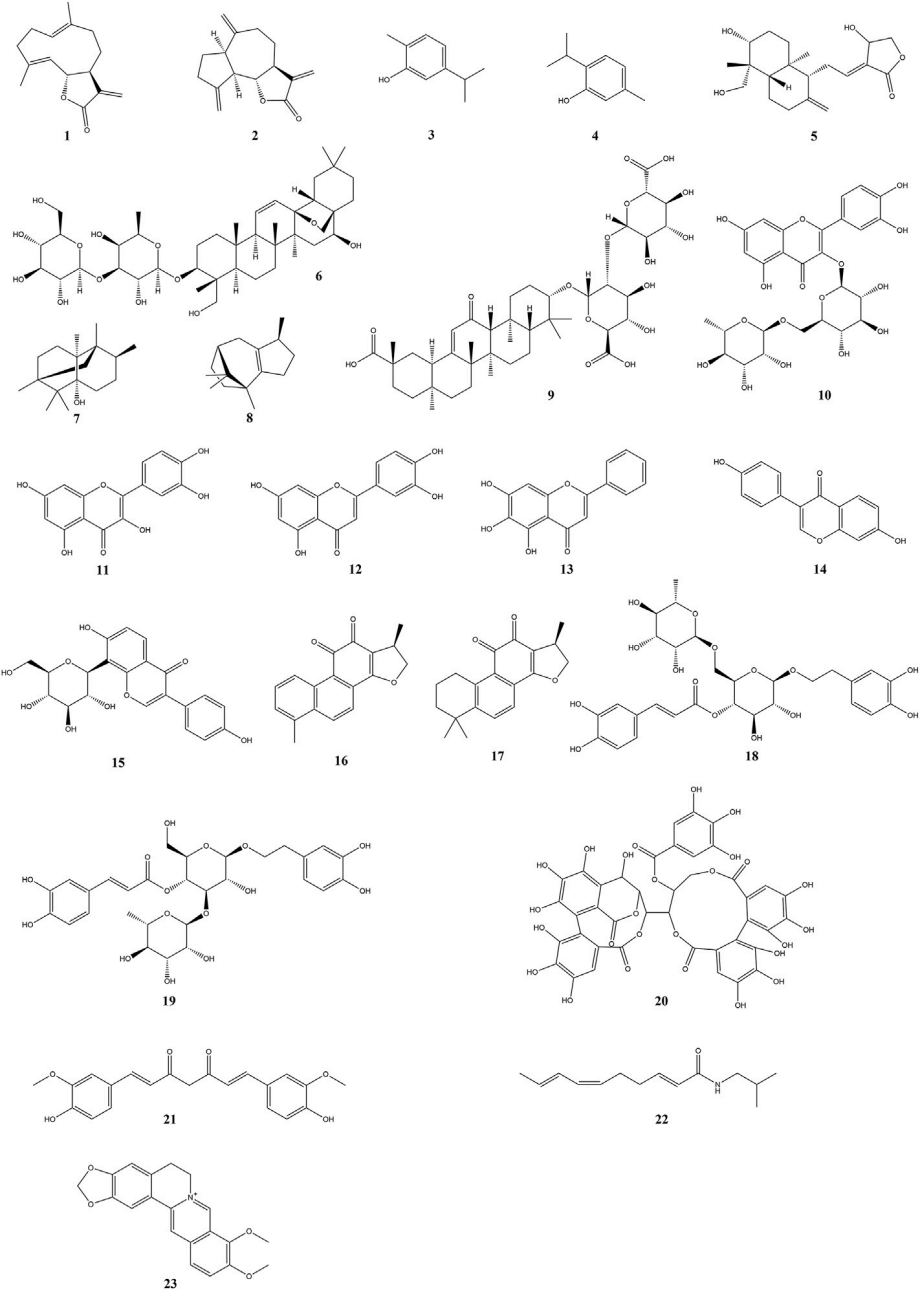
Chemical structures of natural compounds in the treatment of intestinal mucositis.

As is well-known, plants are the most significant natural resources for human survival due to their large variety, quantity, and convenient access. The original sources of human food and drugs were mostly derived from plants. To date, the studies on active constituents have primarily concentrated upon edible and medicinal plants. It is noteworthy that there are plentiful research works on plant-derived natural compounds against intestinal mucositis. In the present research, natural compounds against intestinal mucositis are divided into seven classes, namely, terpenoids, flavonoids, quinones, phenylethanoid glycosides, polyphenols, alkylamides, and alkaloids. The basic, chemical, and pharmacological information of natural compounds used in the therapy of intestinal mucositis is exhibited in Table 1 and Figure 2 and Table 2, respectively.

**TABLE 1:** List of compounds extracted from natural sources.

No.	Compound	Molecular formula	Molecular weight (g/mol)	Main source	Reference
Terpenoids					
1	Costunolide	C_15_H_20_O_2_	232.32	Roots of *Aucklandia lappa*	Chen et al. (2016)
2	Dehydrocostus	C_15_H_18_O_2_	230.3	Roots of *Aucklandia lappa*	Chen et al. (2016)
3	Carvacrol	C_10_H_14_O	150.22	Whole-plant of *Thymus mongolicus* and *Origanum vulgare*	Alvarenga et al. (2016)
4	Thymol	C_10_H_14_O	150.22	Whole-plant of *Thymus vulgaris*, *Origanum vulgare*, and *Ocimum gratissimum*	Al-Khrashi et al. (2021)
5	Andrographolide	C_20_H_30_O_5_	350.45	Aerial part of *Andrographis paniculata*	Xiang et al. (2020); Wang et al. (2022)
6	Saikosaponin A	C_42_H_68_O_13_	780.98	Roots of *Bupleurum chinense* and *Bupleurum scorzonerifolium*	Ali et al. (2019)
7	Patchouli alcohol	C_15_H_26_O	222.366	Aerial part of *Pogostemon cablin*	Wu et al. (2020)
8	β-patchoulene	C_15_H_24_	204.351	Aerial part of *Pogostemon cablin*	Wu et al. (2021)
9	Glycyrrhizic acid	C_42_H_62_O_16_	822.93	Roots and rhizomes of *Glycyrrhiza uralensis*, *Glycyrrhiza inflata*, and *Glycyrrhiza glabra*	Zeeshan et al. (2021)
Flavonoids					
10	Rutin	C_27_H_30_O_16_	610.52	Flowers and fruits of *Sophora japonica*; the whole-plant of *Ruta graveolenslens*	Fideles et al. (2020)
11	Quercetin	C_15_H_10_O_7_	302.24	Flowers of *Sophora japonica*; the leaves of *Platycladus orientalis*; and the rhizomes of *Alpinia officinarum*	Sukhotnik et al. (2018); Lotfi et al. (2021)
12	Luteolin	C_15_H_10_O_6_	286.24	Flowers of *Lonicera japonica* and *Chrysanthemum morifolium* and the aerial part of *Schizonepeta tenuifolia*	Boeing et al. (2020)
13	Baicalein	C_15_H_10_O_5_	270.24	Roots of *Scutellaria baicalensis*	Wang et al. (2020c)
14	Diadzein	C_15_H_10_O_4_	254.24	Roots of *Pueraria lobata*; the seeds of *Glycine max*	Atiq et al. (2019)
15	Puerarin	C_21_H_20_O_9_	416.38	Roots of *Pueraria lobata* and *Pueraria thomsonii*	Wang et al. (2021b)
Quinones					
16	Dihydrotanshinone I	C_18_H_14_O_3_	278.30	Roots and rhizomes of *Salvia miltiorrhiza*	Wang et al. (2020a)
17	Cryptotanshinone	C_19_H_20_O_3_	296.36	Roots and rhizomes of *Salvia miltiorrhiza*	Wang et al. (2020b)
Phenylethanoid glycosides					
18	Forsythiaside A	C_29_H_36_O_15_	624.59	Fruits of *Forsythia suspensa*	Lang et al. (2022)
19	Acteoside	C_29_H_36_O_15_	624.59	Roots of *Rehmannia glutinosa* and the stems of *Cistanche deserticola*	Reinke et al. (2015)
Polyphenols					
20	Casuarinin	C_41_H_28_O_26_	936.65	Roots of *Melastoma malabathricum*	Chen et al. (2022)
21	Curcumin	C_21_H_20_O_6_	368.38	Rhizomes of *Curcuma longa*, *Curcuma aromatica*, *Curcuma zedoaria*, and *Acorus calamus*	Wang et al. (2021b)
Alkylamide					
22	Spilanthol	C_8_H_8_O_3_	152.15	Flowers of *Acmella oleracea*	de Freitas-Blanco et al. (2019)
Alkaloid					
23	Berberine	C_20_H_18_ClNO_4_	371.81	Rhizomes of *Coptis chinensis*, the barks of *Phellodendron chinense*, and the roots of *Berberis soulieana*	Chen et al. (2020a); Yue et al. (2021)

**TABLE 2:** Summary of the mechanisms of natural compounds against intestinal mucositis.

Name	Model	Effective dosage	Molecular mechanism	Reference
Terpenoids				
Costunolide	5-FU-induced intestinal mucositis in Kunming mice	5 and 20 mg/kg	Upregulation: SOD, IL-10, and occludin	Chen et al. (2016)
			Down-regulation: NF-κB, NO, MDA, TNF-α, COX-2, iNOS, and PCNA	
Dehydrocostus	5-FU-induced intestinal mucositis in Kunming mice	5 and 20 mg/kg	Upregulation: occludin	Chen et al. (2016)
			Downregulation: NF-κB, NO, TNF-α, COX-2, iNOS, and PCNA	
Carvacrol	Irinotecan-induced intestinal mucositis in Swiss mice	25, 75, and 150 mg/kg	Upregulation: total leukocytes and NPSH	Alvarenga et al. (2016)
			Downregulation: MPO, TNF-α, IL-1β, KC, NF-κB, COX-2, MDA, and Nox	
Thymol	5-FU-induced intestinal mucositis in Wistar rats	60, 120 mg/kg	Upregulation: GSH, GPx, SOD, and IL-10	Al-Khrashi et al. (2021)
			Downregulation: TBARS, NF-κB, TNF-α, COX-2, IL-6, PGE_2_, p38, p38 MAPK, JNK, p-JNK, and TGF-β	
Andrographolide	5-FU-induced intestinal mucositis in BALB/c mice or NCM460 cells	12.5, 25, 50, and 100 mg/kg *in vivo*	Upregulation: Bcl-2 and ZO-1	Xiang et al. (2020); Wang et al. (2022)
	Irinotecan-induced intestinal mucositis in BALB/c mice or HCT116, HT29, and CT26 cells	0.3, 1, 3, 5, 10, and 20 μM *in vitro*	Downregulation: apoptosis, p-p38/p38, p-p53/p53, Bax, C-Casp8/Casp8, C-Casp3/Casp3, TNF-α, IL-1β, IL-6, p-IRF3, p-TBK1, CD-11b, CD-4, CD-8, Cxcl10, Ccl5, IL-18, IFN-β, dsDNA, γH2AX, and RAD51	
Saikosaponin A	5-FU-induced intestinal mucositis in BALB/c mice	1, 5, and 10 mg/kg	Upregulation: WBC, RBC, lymphocyte, hemoglobin, GSH, GST, CAT, SOD, Nrf2, HO-1, and *Lactobacillus* spp.	Ali et al. (2019)
			Downregulation: granulocyte, hematocrit, MDA, IL-1β, IL-6, TNF-α, NO, p-JNK, COX-2, Casp3, and *Escherichia coli*	
Patchouli alcohol	5-FU-induced intestinal mucositis in SD mice	10, 20, and 40 mg/kg	Upregulation: IL-10, ZO-1, occludin, claudin-1, mucin-2, *Firmicutes*, *S24*, *Allobaculum*, *Erysipelotrichaceae*, and *Peptostreptococcaceae*	Wu et al. (2020)
			Downregulation: TNF-α, IL-1β, IL-6, MPO, TLR2, MyD88, p-IκBα/IκBα, NF-κB p65, MLCK, p-MLC/MLC, *Proteobacteria*, *Bacteroidia*, *ε-Proteobacteria*, *γ-Proteobacteria*, *Phascolarctobacterium*, *Aggregatibacter*, *Helicobacter*, *Pasteurellaceae*, and *Verrucomicrobiaceae*	
β-Patchoulene	5-FU-induced intestinal mucositis in IEC-6 cells and SD rats	10, 20, and 40 mg/kg *in vivo*	Upregulation: mucin-2, goblet cells, claudin-1, and occludin	Wu et al. (2021)
		20 μM *in vitro*	Downregulation: AQP3, PKA, p-MEK1/2/MEK1/2, p-MSK1/MSK1, p-CREB/CREB, VIP, VIPR2, cAMP, PKA, p-MEK1/2/MEK1/2, p-ERK/ERK, p-p38/p38, p-MSK1/MSK1, p-CREB/CREB, P300/CBP, TNF-α, IL-1β, IL-6, IL-10, and p-p65/p65	
Glycyrrhizic acid	5-FU-induced intestinal mucositis in BALB/c mice	10 mg/kg	Upregulation: goblet cells, GSH, GST, and CAT	Zeeshan et al. (2021)
			Downregulation: TNF-α, IL-1β, and IL-6	
Flavonoids				
Rutin	5-FU-induced intestinal mucositis in Swiss mice	50, 100, and 200 mg/kg	Upregulation: GSH and goblet cells	Fideles et al. (2020)
			Downregulation: MPO, MDA, mast cells, and COX-2	
Quercetin	Methotrexate-induced intestinal mucositis in SD rats	5 and 100 mg/kg	Upregulation: BrdU-labeled cells, p-ERK, and CAT	Sukhotnik et al. (2018); Lotfi et al. (2021)
	5-FU-induced intestinal mucositis in albino mice		Downregulation: Casp3, MDA, NF-κB, and HIF-1α	
Luteolin	Irinotecan-induced intestinal mucositis in Swiss mice	3, 10, and 30 mg/kg	Upregulation: GSH, SOD, CAT, IL-4, IL-10, ZO-1, and occludin	Boeing et al. (2020)
			Downregulation: ROS, LOOH, MPO, TNF-α, IL-1β, IL-6, PGE_2_, nitrite, and leukocyte	
Baicalein	5-FU- and irinotecan-induced intestinal mucositis in BALB/c mice	100 mg/kg	Upregulation: *Muribaculaceae*	Wang et al. (2020c)
			Downregulation: IL-6, TNF-α, *Bacteroides*, *Escherichia_Shigella*, *Parabacteroides*, *Enterococcus*, *Clostridium_sensu_stricto_1*, and *Lactococcus*	
Diadzein	5-FU-induced intestinal mucositis in BALB/c mice	1, 5, and 10 mg/kg	Upregulation: mitotic cells, goblet cells, GSH, GST, CAT, WBC, RBC, lymphocytes, platelets, and *Lactobacillus* spp*.*	Atiq et al. (2019)
			Downregulation: TNF-α, IL-6, IL-1β, p-JNK, MDA, nitrite, granulocytes, and *Escherichia coli*	
Puerarin	5-FU-induced intestinal mucositis in C57BL/6 mice or IEC-6 and Caco-2 cells	50 and 100 mg/kg *in vivo*	Upregulation: SOD and GSH	Wang et al. (2021b)
		120 μM *in vitro*	Downregulation: TNF-α, IL-1β, IL-6, COX-2, iNOS, MDA, MLCK, apoptosis, Bax/Bcl-2, p-STAT3/STAT3, SOCS3, p-JAK1/JAK1, p-JAK2/JAK2, p-JAK3/JAK3, and p-TYK2/TYK2	
Quinones				
Dihydrotanshinone I	5-FU- and irinotecan-induced intestinal mucositis in C57BL/6 mice	10 mg/kg	Upregulation: TG, diacylglycerol, *Bacteriodetes*, *Actinobacteria*, *Akkermansia*, *Muribaculaceae*, *Alloprevotella*, *prevotellaceae_UCG_001*, glutamate synthase (NADPH/NADH) small chain, glutamate synthase (ferredoxin), NADH-quinone oxidoreductase subunit F, NADH-quinone oxidoreductase subunit G, and hydroxylamine reductase	Wang et al. (2020a)
			Downregulation: IL-6, TNF-α, *Firmicutes*, *Proteobacteria*, *Lactobacillus*, *Bacteroidetes*, glutathinone metabolism, ABC transporters, purine metabolism, and bacterial invasion of epithelial	
Cryptotanshinone	5-FU- and irinotecan-induced intestinal mucositis in BALB/c mice	20 mg/kg	Upregulation: TG, TG/TC, *Muribaculaceae*, and Ruminococcaceae*_UCG-014*	Wang et al. (2020b)
			Downregulation: IL-6, IL-11, MPO, DAO, TC, and lipase	
Phenylethanoid glycosides				
Forsythiaside A	Methotrexate-induced intestinal mucositis in SD rats	40 and 80 mg/kg	Upregulation: goblet cells	Lang et al. (2022)
			Downregulation: TNF-α, IL-1β, IL-18, leukocytes, neutrophils, lymphocytes, CD68 positive cells, NLRP3, C-Casp1, and cleaved IL-1β	
Acteoside	Methotrexate-induced intestinal mucositis in C57BL/6 mice	600 μg	Downregulation: MPO and MT	Reinke et al. (2015)
Polyphenols				
Casuarinin	5-FU-induced intestinal mucositis in C57BL/6 mice	50 and 100 mg/kg *in vivo*	Upregulation: PCNA^+^ cells, GSH, ZO-1, occludin, *Actinobacteria*, *Lactobacillus murinus*, and *Lachnospiraceae_NK4A136_group*	Chen et al. (2022)
	5-FU-stimulated IEC-6 cells	10, 20, and 30 μM *in vitro*	Downregulation: MDA, MPO, NE, Pr3, CG, IL-1β, TNF-α, *Firmicutes*/*Bacteroidetes*, and *Candidatus Arthromitus*	
Curcumin	5-FU-induced intestinal mucositis in IEC-6 cells	5, 10, and 20 μM	Upregulation: E-cadherin	Wang et al. (2021b)
			Downregulation: apoptosis, TNF-α, IL-1β, IL-6, vimentin, n-cadherin, and p-STAT3	
Alkylamide				
Spilanthol	5-FU-induced intestinal mucositis in Swiss mice	10, 20, and 30 mg/kg	Downregulation: MPO	de Freitas-Blanco et al. (2019)
Alkaloid				
Berberine	5-FU-induced intestinal mucositis in SD rats	50 and 100 mg/kg *in vivo*	Upregulation: occludin, ZO-1, claudin-1, claudin-7, acetate, propionate, butyrate, glutamine, *Firmicutes*, *Porphyromonadaceae*, *Lachnospiraceae*, *Lactobacillus*, *Clostridiales*, *Ruminococcus*, *Prevotella*, and *Clostridium IV*	Chen et al. (2020a); Yue et al. (2021)
	Irinotecan-induced intestinal mucositis in C57BL/6 mice	50 μM *in vitro*	Downregulation: TNF-α, IL-1β, IL-6, IL-8, COX-2, iNOS, LPS, DAO, GUS, *Proteobacteria*, and *Escherichia_Shigella*	
	SN38 stimulated NCM460 or Caco-2 cells			

### Terpenoids

Costunolide and dehydrocostus belong to natural sesquiterpene lactones, which are the main active constituents of *Aucklandia lappa*. Researches have shown that they have anti-inflammatory, antitumor, and antimicrobial activities (Liu et al., 2021). Chen et al. (2016)have investigated the protective actions and possible mechanisms of costunolide and dehydrocostus in 5-fluorouracil-induced gut mucositis. Male Kunming murines were intraperitonelly administered with 5-fluorouracil (60 mg/kg/day) for five days, and intestinal mucositis was assessed based on the histochemical indexes. Costunolide and dehydrocostus were taken orally once a day for eight days. Continuous 5-fluorouracil therapy resulted in serious intestinal mucositis, which was characterized by morphological injury, decreased ingestion, weight loss, and diarrhea. Experimental results showed that the daily gavage of costunolide or dehydrocostus exerted better protective action than that of the positive drug loperamide and prominently alleviated the seriousness of intestinal mucositis *via* accelerating enteric mucosa recovery, reducing ROS level, and restraining the inflammatory reactions. Therefore, costunolide and dehydrocostus are deemed to be promising therapeutical candidates, which are clinically employed to suppress intestinal mucositis in the course of chemical therapy.

Carvacrol is a phenolic monoterpene originating from various essential plants, such as *Thymus mongolicus* and *Origanum vulgare*. *In vitro* and *in vivo* research works have shown that carvacrol has antioxidative, antibacterial, anti-inflammatory, anticancerous, and hepatoprotective effects (Sharifi-Rad et al., 2018). TRPA1 receptor presents high expression in the enteric mucosa and can recognize cell injury, which manifests the potential relation with gut mucositis. Carvacrol is an activator of TRPA1 receptor and possesses anti-inflammatory activity. Therefore, Alvarenga et al. (2016)have conducted studies to prove the assumed anti-inflammatory and protective effects of carvacrol through TRPA1 activization against gut mucositis treated by CPT-11 in murines. In brief, murines were administered with DMSO, CPT-11, or carvacrol prior to CPT-11. In another group, the mice were treated with HC-030031 (TRPA1 antagonist) for thirty minutes prior to carvacrol exposure. On the seventh day, murine survival and microbemia were evaluated, and the jejunal tissues were acquired for morphological analysis and determination of oxidative and inflammatory status after mercy killing. Results indicated that carvacrol exerted an anti-inflammatory effect on CPT-11-treated gut mucositis *via* powerful interplays with TRPA1 receptors, causing the reduction of the levels of pro-inflammatory factors (TNF-α, IL-1β, and KC), other inflammatory mediators (MPO, NF-κB, and COX-2), and oxidant stress (GSH, MDA, and NOx). Carvacrol also facilitated the recovery of the structure of villus and recess in the small intestine and improved clinical indexes, including livability, weight changes, leukocyte, and blood bacteria count. Therefore, carvacrol is a prospective candidate drug for treating gut mucositis, and TRPA1 may be a significant therapeutical target for intestinal mucositis.

Thymol, a natural monoterpenoid phenol, is found in many plants, including *Thymus vulgaris*, *Origanum vulgare*, and *Ocimum gratissimum*. Owing to its various bioactivities, thymol is widely employed in healthcare, animal medicine, food industry, and agriculture. Modern studies have indicated that thymol possesses remarkable beneficial actions, including antimicrobial, antioxidative, anti-inflammatory, anticancer, and immunoregulatory properties (Escobar et al., 2020). Al-Khrashi et al. (2021)have explored the possible protective action and mechanism of thymol on 5-fluorouracil-induced gut mucositis. Murines were injected with 5-fluorouracil (150 mg/kg) and administered with thymol (60 or 120 mg/kg). Pathologic variations, oxidant stress, and inflammatory indicators were evaluated. Results revealed that thymol administration remarkably restrained 5-fluorouracil-induced oxidant stress through decreasing lipid peroxidatic reaction and elevating the intestinal levels of antioxidative systems. Inflammatory indicators, including IL-6, PGE_2_, and COX-2, were also improved. Furthermore, thymol notably suppressed the 5-fluorouracil-induced expression of NF-κB, TNF-α, TGF-β1, p38, p-JNK, and MAPK proteins. Collectively, thymol could alleviate intestinal mucositis *via* the antioxidative and anti-inflammatory roles, which are associated with the inhibition of the TGF-β/p38/p-JNK pathway.

Andrographolide is the main active constituent of *Andrographis paniculata* and possesses a diterpenoid lactone ring. Diversified pharmacological actions of andrographolide have been demonstrated, such as antibacterial, anti-inflammatory, immunoregulatory, antiviral, and anticolitis properties (Kishore et al., 2017). Xiang et al. (2020) probed the protective action and mechanism of andrographolide on 5-fluorouracil-treated gut mucositis. Injection of 5-fluorouracil was conducted for inducing gut mucositis in BALB/c mice. 5-fluorouracil-stimulated NCM460 cells were adopted to probe the potential mechanism of andrographolide *in vitro*. The results indicated that andrographolide markedly relieved 5-fluorouracil-induced weight reduction, diarrhea, and lesions of intestinal crypts and villi. Pro-inflammatory cytokines, including TNF-α, IL-1β, and IL-6, were reduced by andrographolide in colonic and ileal tissues. Moreover, andrographolide significantly inhibited enteral cell apoptosis *in vivo* and *in vitro* through decreasing the expressions of p-p38, p-p53, cleaved caspase-3, cleaved caspase-8, and Bax, and increasing Bcl-2 level. Furthermore, asiatic acid (activator of p38 MAPK) recovered the antiapoptotic effect of andrographolide in NCM460 cells. In addition, Wang et al. (2022)further validated that andrographolide exerted a beneficial role on intestinal mucositis in irinotecan-induced mice and HCT116, CT26, and HT29 cells. Molecular mechanistic studies demonstrated that the andrographolide restrained irinotecan-induced cGAS-STING signaling pathway *via* decreasing the proteic expressions of p-TBK1 and p-IRF3, suppressing the mRNA levels of inflammatory factors (IL-18, TNF-α, and IL-1β), IFN-β, Cxcl10, and Ccl5, and inhibiting the infiltration of immunocytes, including CD11b, CD4, and CD8. The double-stranded DNA release and DNA damage triggered by irinotecan were also mitigated by andrographolide. In conclusion, andrographolide may be beneficial to the sufferers receiving 5-fluorouracil-based chemical therapy.

Saikosaponin-A is a main triterpenoid saponin existed in *Bupleurum falcatum* and *Bupleurum scorzonerifolium*, which manifests anti-inflammatory, antidepressive, antitumor, and immunoregulatory activities (Fu et al., 2015). Ali et al. (2019)demonstrated the protective role of saikosaponin-A on 5-fluorouracil-treated gut mucositis in the murine model. Intestinal mucositis was established in BALB/c murine through injecting 5-fluorouracil for three days, and mucositis was evaluated according to behavior and histochemical analysis. Saikosaponin-A was given one hour prior to 5-fluorouracil treatment for seven days. The results displayed that saikosaponin-A exhibited similar effect to the positive drug mesalazine. Compared with the model group, saikosaponin-A administration notably relieved the severity of intestinal mucositis, including food intake, weight reduction, diarrhea, and death rate in a dosage-dependent manner. The histopathological results also supported the protective action of saikosaponin-A on 5-fluorouracil-induced gut abnormalities, such as villi shrink, crypt stem-cell injury, inflammatory cell infiltration, vacuolation, and edema. Moreover, saikosaponin-A treatment observably suppressed the pro-inflammatory factors (TNF-α, COX-2, IL-1β, and IL-6) and apoptosis indicators (p-JNK and caspase-3). In addition, saikosaponin-A markedly decreased the level of nitric oxide in enteric tissues, restrained acetic acid-treated Evans blue vasopermeability. In addition, siaikosaponin-A administration significantly increased the expressions of antioxidases (Nrf2, HO-1, SOD, GSH, GST, and CAT) and reduced the level of oxidant stress indicator MDA. In brief, the results indicated that saikosaponin-A is a promising drug for treating chemotherapy-induced intestinal mucositis.

Patchouli alcohol, a tricyclic sesquiterpene, is an important active constituent of *Pogostemon cablin*. Up to now, many beneficial effects of patchouli alcohol have been demonstrated, such as anti-inflammatory, antibacterial, anticancer, and anticolitis effects (Lee et al., 2020). Wu et al. (2020) built the murine model of gut mucositis by 5-fluorouracil and administered patchouli alcohol to assess its role on intestinal mucositis. The results indicated that patchouli alcohol manifested similar effect with the positive drug CBL. The general examination (weight, food ingestion, and diarrhea) in animals was adopted to determine the effect of patchouli alcohol on gut mucositis. Inflammatory factors, mucosal barrier proteins, and enteric microbiome were measured to illustrate the potential mechanism of patchouli alcohol against gut mucositis in animals. Experimental results indicated that patchouli alcohol effectually improved weight, food ingestion, and diarrhea in the murine mucositis model, which preliminarily certified the effect of patchouli alcohol. Further tests displayed that patchouli alcohol dramatically reduced the expressions of TNF-α, IL-1β, IL-6, and MPO and elevated the expression of IL-10. Furthermore, the levels of intestinal barrier proteins (e.g., ZO-1, occludin, claudin-1, and mucin-1) and microflora structure were also improved after patchouli alcohol administration in mucositis murines. Therefore, patchouli alcohol may treat intestinal mucositis through decreasing inflammation, sustaining intestinal barrier function, and adjusting gut microbiome.

β-patchoulene, a natural sesquiterpene, is one of the active ingredients in *Pogostemon cablin* (Swamy and Sinniah, 2015). Studies have shown that ß-patchoulene possesses anti-inflammatory, anticolitis, and hepatoprotective effects. Wu et al. (2021) probed the effect and potential mechanism of ß-patchoulene in ameliorating 5-fluorouracil-treated intestinal mucositis in IEC-6 cells and murines. Results indicated that ß-patchoulene had similar effect with the positive drug loperamide, prominently restoring cell activity, increasing body weight and food ingestion, and relieving the pathologic diarrhea symptoms in the gut mucositis rats. Aquaporin is significant for maintaining water fluid homeostasis, and its unusual expression is related to pathological diarrhea in intestinal mucositis. ß-patchoulene exerted a critical role in restraining AQP3 by the cAMP/PKA/CREB signaling pathway. Moreover, inflammation-caused mucus barrier damage destroyed water transport and exacerbated diarrhea in intestinal mucositis animals. The function of ß-patchoulene on restraining inflammation and restoring the mucus barrier reinforced its adjustment of water transport and therefore relieved diarrhea in gut mucositis murines. In conclusion, ß-patchoulene alleviated intestinal mucositis in murines primarily through improving water transport and the mucus barrier, and these activities were related to its role on restraining the cAMP/PKA/CREB signaling pathway.

Glycyrrhizic acid, a triterpene glycoside, is the most significant active constituent of *Glycyrrhiza uralensis*, *Glycyrrhiza inflata*, and *Glycyrrhiza glabra*. Research works have shown that glycyrrhizic acid has anti-inflammatory, antiviral, antioxidative, anticancerous, and immunoregulatory properties (Chen K. et al., 2020). In consideration of the poor pharmacokinetics of glycyrrhizic acid and the prominent therapeutical effect of glycyrrhizic acid-loaded polymeric nanocarriers in intestinal diseases, Zeeshan et al. (2021) investigated their protective role on 5-fluorouracil-induced gut mucositis in murines. Polymeric nanocarrier has been testified to be an effective drug delivery vehicle for the long-range therapy of inflammatory ailments; however, its effect on 5-fluorouracil-induced mucositis has not yet been investigated. Thus, glycyrrhizic acid-loaded polylactic-co-glycolic acid (GA-PLGA) nanoparticle was prepared to assess its preventive and therapeutical effect in 5-fluorouracil-induced mucositis animals. GA-PLGA nanoparticle was produced with an improved double emulsion method, physically and chemically characterized, and tested for *in vitro* drug release. Then, induction of mucositis was conducted by giving 5-fluorouracil to the murines for the first three days, and GA-PLGA nanoparticles were administered to the animals for seven days. GA-PLGA nanoparticle observably decreased the mucositis seriousness concerning body weight, diarrhea scoring, pain, and asitia. Furthermore, 5-fluorouracil caused intestinal histopathologic injury, increased villus-crypt length, decreased goblet-cell quantity, increased pro-inflammatory factors, and restrained antioxidases. Nevertheless, all the deteriorations were restored by GA-PLGA nanoparticle. The results of morphology, behavior, histology, and biochemistry indicated that GA-PLGA nanoparticle was effective, biocompatible, targeted and slow-release nanovehicle drug delivery for enhancing mucoprotective, antiphlogistic, and antioxidative actions in 5-fluorouracil-induced intestinal mucositis.

### Flavonoids

Rutin, also known as vitamin P, is a glycoside of the flavonoid quercetin, derived from many edible and officinal plants, such as *Saussurea involucrata*, *Hippophae rhamnoides*, *Ruta graveolenslens*, and *Sophora japonica*. Extensive research works have shown that rutin possesses antimicrobial, anti-inflammatory, anticancerous, and antidiabetic effects (Ganeshpurkar and Saluja, 2017). Fideles et al. (2020)investigated the function of rutin on 5-fluorouracil-induced experimental gut mucositis. The Swiss murines were randomly separated into seven groups: saline, 5-fluorouracil, rutin-50, rutin-100, rutin-200, celecoxib, and celecoxib + rutin-200 groups. The animals were weighed every day. After therapy, the mice were executed and fragments of the small intestine were gathered to assess the histopathologic changes, the levels of MDA, MPO and GSH, mastocyte and goblet cell amount, and COX-2 vitality. Moreover, the immunohistochemical analysis was also conducted in the study. Rutin administration suppressed 5-fluorouracil-induced histopathologic variations and decreased the oxidant stress through decreasing MDA level and elevating GSH level. Moreover, rutin alleviated the inflammatory reaction *via* reducing MPO vitality, enteric mastocytosis, and COX-2 activity. The result indicated that the COX-2 pathway might be one of the potential protective targets of rutin in treating 5-fluorouracil-induced intestinal mucositis.

Quercetin, a natural flavonoid, isolated from many medicinal and edible plants, such as *Sophora japonica*, *Platycladus orientalis*, and *Alpinia officinarum*. Modern studies have shown that quercetin has antioxidative, anti-inflammatory, antibacterial, and antineoplastic activities (Li et al., 2016). Sukhotnik et al. (2018)assessed the protective effect of quercetin on methotrexate-treated gut injury in SD rats. Results indicated that quercetin notably decreased the enteric damage scores, increased the enteral and mucosal weight of the jejunum and ileum, the protein level in ileum, the length of villi in the ileum, and the depth of crypts in the jejunum and ileum. Moreover, quercetin treatment markedly improved cell proliferation in both the jejunum and ileum through upregulating p-ERK protein expression and downregulating caspase-3. These results indicated that quercetin could restrain gut injury and promote gut restoration after methotrexate-induced gut mucositis. In addition, Lotfi et al. (2021)further found that quercetin and its nanoemulsion formulations exhibited protective effects on 5-fluorouracil-treated gut mucositis in mice. The oxidant–antioxidant balance was regulated by quercetin *via* increasing CAT level and decreasing MDA concentration, and HIF-1α and NF-κB expression.

Luteolin is a usual flavone derived from many kinds of plants, including fruits, vegetables, and Chinese herbal medicines. A great deal of research has shown that luteolin possesses antioxidative, anticancerous, antiallergic, and anti-inflammatory activities (Aziz et al., 2018). Boeing et al. (2020)investigated the role and mechanism of luteolin against irinotecan-treated gut mucositis in murines. Clinical symptoms, histologic, oxidized, and inflammatory indicators were examined, along with the potential effect of luteolin on the antineoplastic role of irinotecan. The results indicated that luteolin improved irinotecan-induced gut injury through decreasing weight loss and diarrhea scores and suppressing the shortening of the dodecadactylon and colon. Histologic examination proved that luteolin restrained villi shortening, vacuolation, and cell apoptosis and maintained the mucoprotein level in the dodecadactylon and colon. Furthermore, luteolin administration alleviated irinotecan-induced oxidant stress through decreasing the expressions of ROS and LOOH and increasing endogenic antioxygens and suppressed inflammatory symptoms through reducing MPO vitality, TNF-α, IL-1β, IL-6 productions, and elevating IL-4 and IL-10 productions. Disruption of the tight junctions ZO-1 and occludin could also be reversed by luteolin administration. It is noteworthy that luteolin had no effect on the anticancer role of irinotecan. In brief, luteolin may be a promising candidate to inhibit intestinal mucositis caused by irinotecan.

Baicalein, a natural flavone, is predominantly found in the roots of *Scutellaria baicalensis*. It has been proven that baicalein possesses antioxidative, antiviral, antibacterial, anti-inflammatory, antiallergenic, and antitumor effects (Dinda et al., 2017). Wang et al. (2020c) estimated the therapeutical effect of baicalein against chemotherapy-induced gut mucositis in murines. Simultaneously, the role of enteric microflora modulation in the therapeutical action of baicalein was explored. Male BALB/c murines were randomly separated into three groups, namely, the normal control, model, and experimental groups. Apart from normal control group, murines were treated with 5-fluorouracil and irinotecan to establish the intestinal mucositis model. Baicalein notably decreased the DAI of intestinal mucositis animals and the levels of IL-6 and TNF-α in serum. There were remarkable discrepancies in the constitution of the intestinal microflora among groups based on the analyses of *a*-diversity, ß-diversity and the species discrepancies. In comparison with the normal group, the *Ruminococcaceae_UCG_014* and *Lachnospiraceae* in murines of the model group were markedly reduced, while *Bacteroides*, *Escherichia_Shigella*, *Enterococcus*, *Parabacteroides*, *Clostridium_ sensu_stricto_1*, and *Lactococcus* were observably elevated. After baicalein treatment, the levels of *Bacteroides*, *Escherichia_Shigella*, *Parabacteroides*, *Enterococcus*, *Clostridium_sensu_stricto_1*, and *Lactococcus* were notably reduced and *Muribaculaceae* was elevated. The levels of IL-6 and TNF-α in the serum of the three groups were positively related to the levels of *Clostridium_sensu_stricto_1*, *Lactococcus*, *Bacteroides*. and *Enterococcus* based on the association analysis. The present research indicated the promising efficacy of baicalein on intestinal mucositis murines. Modulation of intestinal microflora possibly exerted a crucial role in the therapeutic effect of baicalein.

Diadzein is a natural isoflavone mainly extracted from *Pueraria lobata* and soybeans. Extensive pharmacologic actions of diadzein were proven, including anticancer, anti-inflammatory, and antidiabetic activities (Feng et al., 2015). Atiq et al. (2019)investigated the effect of diadzein on gut mucositis treated with 5-fluorouracil and payed close attention to oxidant stress and inflammatory indicators in murines. Animals were given 5-fluorouracil to induce mucositis once daily for three days, and diadzein was given once daily for seven days. The results showed that diadzein exerted a comparable effect with the positive drug mesalazine. Administration of diadzein decreased the seriousness of mucosa damage in a dosage-dependent manner. Diadzein prominently suppressed weight reduction, alleviated diarrhea, and improved histopathologic deformity related to inflammatory symptoms. Furthermore, diadzein observably improved the enteric wall histopathology through decreasing infiltration and arrested restraint of antioxygen *via* 5-fluorouracil treatment. Nitrite levels in enteral tissues were decreased by diadzein, which accorded with the regulation of inflammatory mediators. In addition, diadzein also improved the microbial profile through decreasing the count of pathogens and increasing the level of probiotic bacteria. Collectively, this research indicated that diadzein possessed prominent protective effect against mucositis in 5-fluorouracil-treated mucositis model, and the therapeutical effect of diadzein may be associated with the restraint of oxidant stress and inflammatory response.

Puerarin, a natural isoflavone, is the major constituent derived from the roots of *Pueraria lobata*. Research works have shown that puerarin has antitumor, antiviral, antioxidative, and anti-inflammatory effects (Zhang, 2019). Wang L. et al. (2021) built a murine model of gut mucositis through intraperitoneally injecting 5-fluorouracil and then administrating puerarin for seven days. Regular indices including weight, food intake, and diarrhea were measured to assess the effect of puerarin on gut mucositis in murines. The function of gut barrier was also assessed through determining the serum recovery of fluorescein isothiocyanate-4kD dextran. The expressions of inflammatory factors, oxidative reactions, and apoptosis marker proteins were examined to illuminate the potential mechanism of puerarin against gut mucositis. Results suggested that the model murines appeared representative symptoms and histopathologic variations of 5-fluorouracil-induced gut mucositis. Drastic inflammatory symptoms, oxidation reactions, cell apoptosis, and JAK were significantly stimulated. Puerarin reduced the expressions of inflammation-related proteins, oxidation reactions and apoptosis in 5-fluorouracil-induced mucositis through blocking JAK activation. In conclusion, puerarin reduced inflammatory symptoms, oxidation reactions and apoptosis, and protected enteric barrier function to alleviate 5-fluorouracil-treated gut mucositis *via* suppressing JAK activation. This work suggested that puerarin might be a promising natural JAK depressor for the treatment of 5-fluorouracil-induced intestinal mucositis.

### Quinones

Dihydrotanshinone I is an important active constituent in *Salvia miltiorrhiza*, which has been reported to exert antitumor, anti-inflammatory, and cardioprotective roles (Chen et al., 2019). Wang et al. (2020a) probed the action of dihydrotanshinone I against intestinal mucositis induced by 5-fluorouracil and irinotecan in murines. They determined the level of enteric mucosa injury and inflammatory reaction in gut mucositis animals with or without dihydrotanshinone I treatment. Body weight and DAI of murines were daily examined. H&E staining was adopted to assess the pathologic injury. The levels of IL-6, TNF-α, TG, and diacylglycerol in serums were measured using commercial kits. The variations of fecal microbiome were also probed *via* 16S rRNA high throughput sequencing. Spearman association analyses were employed to assess the relevance between fecal microbiome and inflammatory cytokines. Tax4Fun was used to explore the possible role of gut microflora. The result indicated that dihydrotanshinone I markedly decreased the DAI score, enteric mucosa injury, and inflammatory reaction in gut mucositis murines by reducing the serum IL-6 and TNF-α levels. Moreover, there was close relationship between fecal microbiome and inflammatory cytokines. Dihydrotanshinone I effectively restored the maladjusted faecal microbiome and increased the level of *Akkermansia*. Dihydrotanshinone I also improved bacteria species, which facilitated butyric acid metabolism or were negatively related to inflammatory cytokines. In addition, species improved by dihydrotanshinone I in faecal microbiome were possibly associated with the glutamine level and ammonia oxidization. In brief, this research indicated that dihydrotanshinone I effectually alleviated gut mucositis caused by 5-fluorouracil and irinotecan in murines. Adjustment of the composition and function of fecal microbiome possibly exerted an important effect in the therapeutical action of dihydrotanshinone I in intestinal mucositis animals.

Cryptotanshinone is a natural quinone compound predominantly existed in *Salvia miltiorrhiza*. Studies have indicated that cryptotanshinone has anti-inflammatory, anticancerous, antibacterial, neuroprotective, and cardioprotective effects (Li et al., 2021). Wang et al. (2020b) explored the therapeutical action of cryptotanshinone on chemotherapy-induced gut mucositis in murines. Chemotherapy-caused gut mucositis was induced through intraperitoneally injecting 5-fluorouracil and irinotecan for four days. A pseudo-sterile murine model was established through intragastrically administering mixed antibiotics (metronidazole, vancomycin, and penicillin). Weight, DAI, and fasces of murines were daily examined and the levels of inflammatory cytokines, TC, TG, and lipase vitality in serums or colon mucosa of intestinal mucositis animals were measured. They also probed the constitution and relative abundance of fecal microflora. The relation of the relative abundance of fecal microflora and environmental aspects was further analyzed. Cryptotanshinone observably reduced DAI, and the expressions of IL-6, IL-11, MPO, and DAO in the serums of intestinal mucositis murines. Cryptotanshinone effectually elevated the level of TG while decreased TC and lipase vitality in the serums. It was indicated that the incidence of chemotherapy-induced mucositis in the pseudoaseptic model group was prominently decreased. In the meantime, there was no remarkable discrepancy in the levels of inflammatory cytokines and TG/TC ratio between the pseudoaseptic model and normal control groups. There was a prominent discrepancy in the diversity and constitution of fecal microbiome among groups. Furthermore, cryptotanshinone recovered the constitution of fecal microbiome close to normal and observably elevated the levels of *Muribaculaceae* and *Ruminococcaceae_UCG-014*. Specially, *Ruminiclostridium* and *Muribaculaceae* showed a remarkable positive relation to TG but a negative relation to DAO, MPO, IL-6, lipase, and TC. In brief, cryptotanshinone effectually relieved gut mucositis in murines induced by 5-fluorouracil and irinotecan through modulating the fecal microbiome, inflammatory cytokines, and serum lipids.

### Phenylethanoid glycosides

Forsythiaside A is the major active constituent derived from *Forsythia suspensa* and has excellent biological activities. Pharmacological research works have proven that forsythiaside A has remarkable activities in treating multiple diseases, including inflammation, virus and bacterial infection, oxidative stress, and hepatic damage (Gong et al., 2021). Lang et al. (2022) explored the effect and mechanism of forsythiaside A on chemotherapy-induced gut mucositis in murines. In brief, for three days, male SD murines were treated with 7 mg/kg methotrexate to induce intestinal mucositis and simultaneously administered with 40 or 80 mg/kg forsythiaside A for seven days. The result indicated that the final body weight and daily food uptake were elevated, and the DAI score was decreased by forsythiaside A administration. The methotrexate-treated rats exhibited pathologic changes including inflammatory infiltration, mucosa layer destruction, gland expansion, enteric villus structure disorder, and goblet cell reduction, whereas administration with 80 mg/kg forsythiaside A showed obvious beneficial roles. ELISA assay further indicated that the serum levels of TNF-α, IL-1β, and IL-18 in methotrexate-induced animals were decreased after forsythiaside A administration. Furthermore, forsythiaside A reduced the amounts of leukocytes, neutrophils, and lymphocytes in peripheral blood. Western blotting and immunofluorescence results showed that the expressions of NLRP3, cleaved caspase 1 and cleaved IL-1β and CD68 positive rate were reduced in methotrexate-induced murines after forsythiaside A treatment. In conclusion, forsythiaside A effectively restrained methotrexate-induced intestinal mucositis through alleviating the activation of NLRP3 inflammasome.

Acteoside is an important active component, which mainly exists in medicinal plants like *Rehmannia glutinosa* and *Cistanche deserticola*. At present, acteoside has been found to exert anti-inflammatory, antioxidative, and anticancer effects (Zhou et al., 2020). Reinke et al. (2015) explored the effect of acteoside in the treatment of intestinal mucositis. C57BL/6 murine was administered daily with 600 μg acteoside for five days before inducing intestinal mucositis and throughout the experiment. Induction of intestinal mucositis was conducted by methotrexate, and mice were executed on the fifth and eleventh days after methotrexate. The dodecadactylon, jejunum, and ileum were collected to examine MPO vitality, MT expression, and histological features. Acteoside decreased histological severity scores by 75, 78, and 88% in the dodecadactylon, jejunum and ileum in comparison with the methotrexate controls on the fifth day, respectively. Acteoside also decreased crypt depth by 49, 51, and 33% and elevated villus height by 19, 38, and 10% in the dodecadactylon, jejunum, and ileum in comparison with the methotrexate controls on the fifth day, respectively. Moreover, acteoside reduced the MT level by 50% in comparison with the methotrexate control murine on the fifth day. Moreover, acteoside reduced the MPO level by 60 and 30% in the dodecadactylon and jejunum in comparison with the methotrexate controls on the fifth day, respectively. The results indicated that acteoside relieved methotrexate-induced intestinal mucositis probably through suppressing inflammation.

### Polyphenols

*Melastoma malabathricum* is a common herb and mainly adopted to treat multiple gastrointestinal diseases in China (Al-Sayed et al., 2020). Chen et al. (2022) isolated an active compound casuarinin from the root of *Melastoma malabathricum* and explored the therapeutic effect and mechanism of casuarinin in 5-fluorouracil-treated gut mucositis murines. Results indicated that casuarinin notably improved 5-fluorouracil-induced body weight loss and ingestive reduction, and also prominently reversed villus atrophy, restored the proliferative activity of the enteric crypts, and restrained inflammatory symptoms and enteric barrier dysfunction in the murine model of 5-fluorouracil-treated gut mucositis. Moreover, casuarinin also reversed 5-fluorouracil-induced intestinal microflora dysbiosis, especially the level of *Actinobacteria*, *Candidatus Arthromitus*, *Lactobacillus murinus*, and the Firmicutes-to-Bacteroidetes ratio. In conclusion, casuarinin could improve 5-fluorouracil-treated gut mucositis by favorably regulating inflammation, gut barrier dysfunction, and intestinal microflora imbalance. Therefore, casuarinin might be a prospective candidate for the treatment of intestinal mucositis.

Curcumin, a natural diketone, mainly exists in *Curcuma longa*, *Curcuma aromatica*, *Curcuma zedoaria*, and *Acorus calamus*. For a long time, curcumin has been widely used in the food industry as a common natural pigment, and also exhibits antioxidative, antibacterial, antiviral, antitumor, and anti-inflammatory activities (Hewlings and Kalman, 2017). Considering the anticancer effect of curcumin and its protective effect in intestinal diseases, Wang et al. (2021)explored the role of curcumin in inflammation and enteric epithelial cell injury in gut mucositis animals. 5-fluorouracil was adopted to induce intestinal mucositis in gut epithelia cells, and diverse levels of curcumin were given. Experimental data indicated that curcumin effectively alleviated 5-fluorouracil-induced injury to IEC-6 cells, restrained the expressions of inflammatory factors, increased cell vitality, and exerted antiapoptotic role on IEC-6 cells. RNA sequencing analyses and experimental verification demonstrated that curcumin exerted protective effect on 5-fluorouracil-induced gut mucositis in enteral epithelia cells through inhibiting the IL-6/STAT3 signaling pathway. In conclusion, the results manifested that curcumin may be a potential therapeutical candidate in preventing and treating chemotherapy-induced intestinal mucositis.

### Alkylamide

Spilanthol, a major alkylamide from *Acmella oleracea*, possesses narcose, analgesic, and anti-inflammatory actions (Silveira et al., 2018). de Freitas-Blanco et al. (2019)assessed the effect of spilanthol on gut mucositis in Swiss murine elicited by 5-fluorouracil. Continuous dose of 5-fluorouracil led to gut mucositis and consequently decreased food ingestion and weight reduction. Experimental data suggested that spilanthol (30 mg/kg) prominently reduced the seriousness of gut mucositis, restrained histopathologic deterioration, and increased the villi length in mice administered with spilanthol compared with the 5-fluorouracil treatment group. Though a few pro-inflammatory factors were not quantifiable in any group, reduction of MPO vitality was obvious in mice administered with spilanthol. In brief, the results indicated that spilanthol efficiently decreased the inflammatory symptoms in gut mucositis mice treated with 5-fluorouracil, and this natural compound may be a potential therapeutical candidate for gut mucositis.

### Alkaloid

Berberine is a natural isoquinoline alkaloid existed in a lot of medicinal plants including *Coptis chinensis*, *Phellodendron chinense*, and *Berberis soulieana*. Modern pharmacological studies have shown that berberine possesses hypoglycemic, hypolipidemic, antibacterial, and anti-inflammatory activities (Kumar et al., 2015). Chen H. T. et al. (2020)explored the function and mechanism of berberine on gut mucositis rats treated by 5-fluorouracil. Results indicated that berberine treatment exhibited decreased weight loss, reduced diarrhea score, and increased colon length in 5-fluorouracil-induced gut mucositis rats. Meanwhile, berberine dramatically increased the occludin level in ileum and lowered the d-lactic acid content in serum. In addition, berberine prominently restrained inflammatory response through inhibiting the production of intestinal inflammatory cytokines TNF-α, IL-6, and IL-1β. Moreover, berberine reversed the changes of fecal metabolites and regulated metabolic pathways via increasing the levels of acetate, butyrate, propionate, and glutamine. Importantly, berberine effectively modulated the gut microbiota structure. The relative abundance of *Firmicutes*, *Porphyromonadaceae*, *Lachnospiraceae*, *Lactobacillus*, *Clostridiales*, *Ruminococcus*, *Prevotella*, and *Clostridium IV* was upregulated, while *Proteobacteria* and *Escherichia_Shigella* were decreased by berberine treatment. Significantly, fecal transplantation from berberine-treated rats could relieve intestinal mucosal injury. Yue et al. (2021)also found that berberine conspicuously ameliorated gut mucositis in irinotecan-treated mucositis murines and SN38-stimulated NCM460 or Caco-2 cells. The underlying mechanism might be closely associated with the suppression of pro-inflammatory mediators (including COX-2, iNOS, TNF-α, IL-8, and IL-1β), bacterial GUS activity and the improvement of mucosal barrier integrality by elevating the protein expression of ZO-1, occludin, and claudin-1 and decreasing the levels of LPS, DAO, and FITC-dextran fluorescence. In summary, these results offered novel clues into the potential usage of berberine in preventing and treating intestinal mucositis.

## Discussion

Chemotherapy for malignant tumors can cause changes in the structure and function of the gastrointestinal tract. 5-fluorouracil, an important chemotherapeutic drug, is widely used to treat advanced colorectal carcinoma and malignant tumors in head and neck (Cheah et al., 2014b; Huang et al., 2022). Consecutive oral administration of 5-fluorouracil leads to serious toxic reactions, especially gut mucositis, which is mainly ascribed to the destruction of intestinal structure and integrity. Moreover, epithelial cell apoptosis, oxidative stress, inflammatory response, and intestinal microflora disorder are closely associated with the deterioration of intestinal mucositis.

Intestinal barrier is composed of epithelia barrier and mucosal layer. The intact mucosal layer is a barrier to stop water runoff and eliminate inspiratory foreign matters, including microorganisms, inflammatory cells, and pollutants (Bajka et al., 2015; Zhao and Maynard, 2022). When intestinal mucositis appeared, slime layer is exhausted with reduced level of mucins (mucin-1 and mucin-2) and goblet cell dysregulation (Kawashima et al., 2015; Thorpe et al., 2020). Malfunction of the mucus layer, in turn, exacerbated inflammatory symptoms and impaired the water-holding capability of the gastrointestinal tract, caused aquaporins over expression, and ultimately resulted in serious diarrhea in intestinal mucositis murines (Ikarashi et al., 2016). In the current review, patchouli alcohol, ß-patchoulene, glycyrrhizic acid, rutin, and forsythiaside A could effectively improve the content of goblet cells or mucins. Of note, ß-patchoulene enhanced the mucus layer function and decreased aquaporin 3 level, thus recovering the murine’s water-holding capability. The association analysis also showed that aquaporin 3 and diarrhea symptoms were strongly associated with mucus layer function.

Intestinal epithelia establish the maximal and most significant barrier between inner and outer surroundings. Research works have indicated that enteric epithelium barrier is destroyed in gastrointestinal disorders, such as ulcerative colitis and gut mucositis (Xiong et al., 2019; Jin et al., 2022). TJs are the apical adhesive junction complexes in epithelial cells of mammals. Deficiency of TJs results in an enhancement in enteric penetrability and promotes the development of enteric inflammation (Panwar et al., 2021). TJ membrane-spanning proteins, such as ZO-1, ZO-2, occludin, and claudins, exert a synergic action in controlling the entry and exit of ionic solute and maintaining enteric penetrability. ZO is a kind of cytoplasmic scaffolding protein, which anchors TJ membrane protein to the cytoskeleton through interaction (Skamrahl et al., 2021). Claudins are the critical constituents of intercellular TJs and take charge of paracytic solute flux (Tanaka et al., 2015). As a necessary membrane-spanning protein, occludin is significant for TJ integrity. In addition, MLCK exerts a key effect in TNF-α-induced enteric penetrability. MLCK restraint stops MLC phosphorylation and maintains enteric TJ barrier, leading to the inhibition of enteric inflammation (Wang J. et al., 2021). Current research works have found a destruction of the enteric intercellular structure, with an increase in enteric penetrability, reduced TJs levels, and abnormal high expressions of MLC, MLCK and p-MLC proteins in mucositis murines. However, patchouli alcohol, ß-patchoulene, luteolin, casuarinin, and berberine exhibited beneficial action on enteric penetrability by elevating the levels of TJs (ZO-1, claudin-1, or occludin), thus resulting in further maintaining enteric epithelia barrier. Moreover, the increased level of MLCK and p-MLC were significantly inhibited by puerarin and patchouli alcohol treatments.

Inflammatory infiltration is a typical feature of mucositis. MPO vitality and pro-inflammatory factors have been found to increase under inflammatory conditions (Li et al., 2020). Excessive production of ROS and uncontrolled inflammatory symptoms could disturb enteric homeostasis, change the normal microflora and stimulate a sequence of acute reactions to injure cells and tissues, resulting in mucosa damage and mucositis (Boeing et al., 2020). The content of antioxidases decreases or cannot remove superfluous ROS, resulting in elevated oxidative stress and cell injury (Yoneda et al., 2021). Moreover, convincing evidence have demonstrated that elevated expressions of inflammatory factors were strongly related to the destruction of enteric mucosa barrier (Tappenden, 2008). Research works have indicated that 5-fluorouracil treatment could elevate the expressions of pro-inflammatory cytokines (COX-2, iNOS, IL-6, IL-1β, and TNF-α), reduce antioxidant enzymes (SOD, GST, and CAT), and induce mucosa injury (Aghabozorgi et al., 2020). However, costunolide, thymol, saikosaponin A, rutin, luteolin, and casuarinin have been proven to possess anti-inflammatory action and relieve oxidative stress by reducing the generation of pro-inflammatory factors and elevating the expressions of antioxidants.

TLRs possess critical effects in adjusting the enteric epithelia barrier and congenital immunization. TLR2 induce NF-κB activation to regulate the gene levels of inflammatory mediators by Toll/IL-1 receptor domain with adapter protein MyD88 (Takeuchi and Akira, 2002; Zhang et al., 2021). Proper expression of TLR2 insures antiapoptotic action, resulting in the adjustment of TJs in enteric epithelia cells (Lin et al., 2013). NF-κB is a significant coordinator of congenital and adaptive immunoreactions, taking part in the regulation of inflammatory symptoms and cell cycle. NF-κB activation induces the abnormal expression of its downstream genes, including iNOS, COX-2, chemokines, adhesion molecules, and pro-inflammatory factors (Luqman and Pezzuto, 2010). In addition, STAT3 is a cellular signal transcription factor, which plays a key role in hyperplasia, differentiation, transference, and survival. Enduring STAT3 activation is deemed to accelerate chronic inflammation, which elevates susceptibility of healthy cells to carcinogenesis (Lei et al., 2021). When the enteric mucosa is activated, increased IL-6 can stimulate the phosphorylation of STAT3 and feedback to NF-κB (Ke et al., 2015). In the present review, patchouli alcohol, thymol, puerarin, and forsythiaside A remarkably restrained intestinal mucositis in animal or cell models, which was intimately associated with the suppression of TLR2/MyD88/NF-κB, IL-6/STAT3, TGF-β/MAPK, and NLRP3 signaling pathways.

Intestinal microbiota homeostasis is a significant element for gastrointestinal health. Numerous research works have shown that gut microflora disorder exhibits a crucial effect in gastrointestinal diseases, including colitis, bacterial dysentery, and chemotherapy-induced mucositis (Varankovich et al., 2015). Microbial diversity analysis has shown that compared with the normal group, the intestinal microbial structure of mucositis animals changed significantly. Moreover, microflora takes part in the change of inflammatory cell factors (Li et al., 2017). *Bacteroides*, *Bifidobacterial*, *Lactobacilli*, *Escherichia*, *Helicobacter*, and *Parabacteroides* are strongly associated with the progression and seriousness of mucositis. *Bifidobacterial* and *Lactobacilli* are indicated to be capable of restraining NF-κB activation, increasing TJs protein level, and reducing enteric penetrability (Khokhlova et al., 2012; Wu et al., 2016). *Bacteroides*, *Escherichia*, *Helicobacter*, and *Parabacteroides* are often deemed to be the pathogenic bacteria or conditioned pathogens (Zhang et al., 2018). For instance, rises in *Parabacteroides* and *Bacteroides* amounts contribute to colitis in murines, *Helicobacter pylori* infection contributes to peptic ulcer disorder and stomach tumors, and *Escherichia* is profitable for gut inflammation *in vivo*. *Bifidobacterium infantis* alleviates Th1 cell reaction through adjusting cell factors and differentiation-related factors in chemotherapy-induced gut mucositis murines (Mi et al., 2017). In terms of *Lactobacillus*, it contributes to reducing the colonic pH and the development of pathogens (Yeung et al., 2020). In this review article, 5-fluorouracil induced structure disorder of intestinal microflora characterized by elevated *Bacteroides*, *Escherichia coli*, *Proteobacteria*, *Bacteroidia*, *Proteobacteria*, *Helicobacter*, *Pasteurellaceae*, *Verrucomicrobiaceae*, and reduced relative abundances of *Bacteriodetes*, *Lactobacillus* spp., *Actinobacteria*, and *Lactobacillus murinus*, which were reversed by saikosaponin A, patchouli alcohol, baicalein, daidzein, dihydrotanshinone, casuarinin, and berberine treatment.

## Conclusion

**FIGURE 3:**
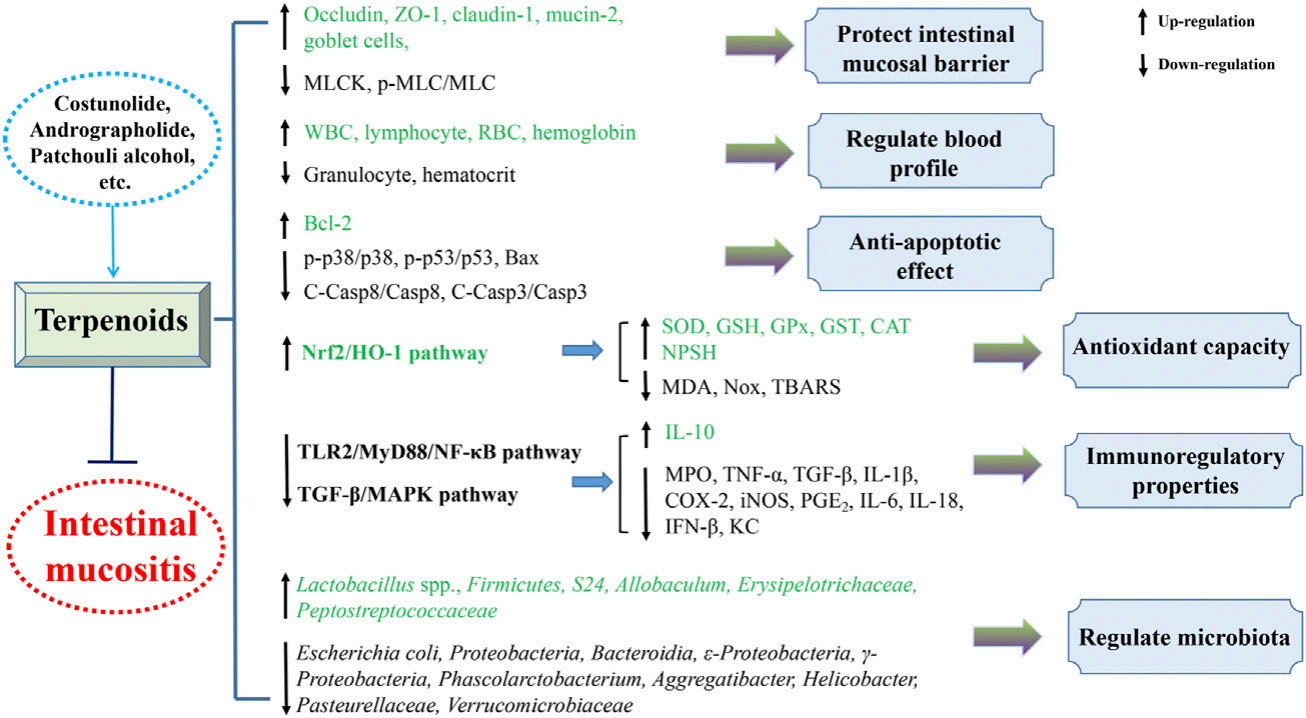
Mechanism of natural terpenoids in the therapy of intestinal mucositis.

**FIGURE 4:**
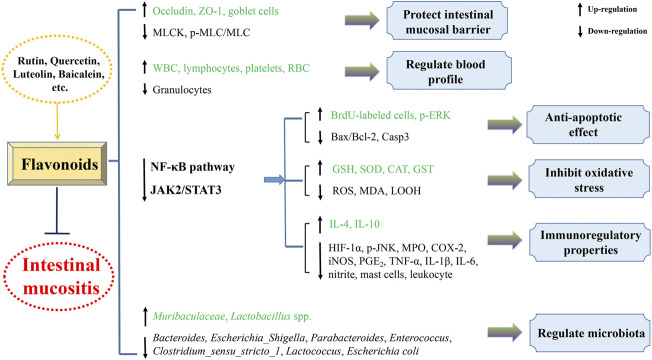
Mechanistic diagram of natural flavonoids in the treatment of intestinal mucositis.

The present review generalizes the significant and beneficial roles of plant-derived natural compounds in attenuating intestinal mucositis in pioneering endeavor. Their protective mechanisms against intestinal mucositis might involve improving intestinal barrier function, decreasing enterocyte apoptosis, regulating gut microbiota, and inhibiting inflammatory reaction and oxidant stress predominantly through blocking TLR2/MyD88/NF-κB, TGF-β/MAPK, JAK2/STAT3, Nrf2/HO-1, and NLRP3 inflammasome pathways (Figures 3, 4). Taken together, these findings provide experimental and scientific evidence for utilizing plant-derived natural compounds as drug candidates to treat intestinal mucositis. However, further in-depth preclinical and clinical trials are warranted to clarify the molecular mechanism to facilitate the potential clinical transformation of natural compounds like costunolide, andrographolide, patchouli alcohol, and berberine in the treatment of intestinal mucositis.

## Glossary

AQP3: aquaporin 3
CAT: catalase
CBL: live combined bifidobacterium
C-Casp: cleaved caspase
CG: cathepsin G
COX-2: cyclooxygenase-2
CPT-11: irinotecan hydrochloride
CREB: element-binding protein
DAI: disease activity index
DAO: diamine oxidase
DMSO: dimethylsulfoxide
5-FU: 5-fluorouracil
GPx: glutathione peroxidase
GSH: glutathione
GST: glutathione S-transferase
GUS: ß-glucuronidase
HIF-1α: hypoxia-inducing factor 1α
HO-1: hemoxygenase-1
IFN: interferon
iNOS: inducible nitric oxide synthase
IL: interleukin
JAK: janus kinase
KC: keratinocyte chemoattractant
LOOH: lipid hydroperoxides
LPS: lipopolysaccharide
MAPK: mitogen-activated protein kinase
MASCC/ISO: mucositis study group of the multinational association of supportive care in cancer/international society of oral oncology
MDA: malondialdehyde
MLC: myosin light chain
MLCK: myosin light chain kinase
MPO: myeloperoxidase
MT: metallothionein
NF-κB: nuclear factor kappa-B
NE: neutrophil elastase
NOx: nitrogen oxide
NPSH: non-protein sulfhydryl
Nrf2: nuclear factor erythroid 2-related factor 2
PCNA: proliferating cell nuclear antigen
p-ERK: phosphorylated extracellular signal-related kinase
p-IRF3: phosphorylated interferon regulatory factor 3
PGE2: prostaglandin E2
p-JNK: phosphorylated c-Jun N terminal kinase
PKA: protein kinase A
Pr3: proteinase 3
p-STAT3: phosphorylated signal transducer and activator of transcription
p-TBK1: phosphorylated TANK-binding kinase 1
p-TYK: phosphorylated TYK
ROS: reactive oxygen species
SD: Sprague–Dawley
SOCS3: suppressor of cytokine signaling 3
SOD: superoxide dismutase
STAT3: signal transducers and activators of transcription 3
TBARS: thiobarbituric acid reactive substances
TC: total cholesterol
TG: triglyceride
TJ: tight junction
TNF: tumor necrosis factor
TRPA1: transient receptor potential cation channel, subfamily A, member 1
TYK: tyrosine kinase
Tyr: tyrosinase
ZO-1: zonula occludens

## References

[B1] Aghabozorgi A. S. Sarabi M. M. Jafarzadeh-Esfehani R. Koochakkhani S. Hassanzadeh M. Kavousipour S. (2020). Molecular determinants of response to 5-fluorouracil-based chemotherapy in colorectal cancer: The undisputable role of micro-ribonucleic acids. World J. Gastrointest. Oncol. 12 (9), 942–956. 10.4251/wjgo.v12.i9.942 33005290 PMC7510001

[B2] Al-Khrashi L. A. Badr A. M. Al-Amin M. A. Mahran Y. F. (2021). Thymol ameliorates 5-fluorouracil-induced intestinal mucositis: Evidence of down-regulatory effect on TGF-β/MAPK pathways through NF-κB. J. Biochem. Mol. Toxicol. 36 (1), e22932. 10.1002/jbt.22932 34665902

[B3] Al-Sayed E. Michel H. E. Khattab M. A. El-Shazly M. Singab A. N. (2020). Protective role of casuarinin from *Melaleuca leucadendra* against ethanol-induced gastric ulcer in rats. Planta Med. 86 (1), 32–44. 10.1055/a-1031-7328 31689719

[B4] Ali J. Khan A. U. Shah F. A. Ali H. Ul Islam S. Kim Y. S. (2019). Mucoprotective effects of Saikosaponin-A in 5-fluorouracil-induced intestinal mucositis in mice model. Life Sci. 239, 116888. 10.1016/j.lfs.2019.116888 31639401

[B5] Alvarenga E. M. Souza L. K. M. Araujo T. S. L. Nogueira K. M. Sousa F. B. M. Araujo A. R. (2016). Carvacrol reduces irinotecan-induced intestinal mucositis through inhibition of inflammation and oxidative damage *via* TRPA1 receptor activation. Chem. Biol. Interact. 260, 129–140. 10.1016/j.cbi.2016.11.009 27838229

[B6] Atiq A. Shal B. Naveed M. Khan A. Ali J. Zeeshan S. (2019). Diadzein ameliorates 5-fluorouracil-induced intestinal mucositis by suppressing oxidative stress and inflammatory mediators in rodents. Eur. J. Pharmacol. 843, 292–306. 10.1016/j.ejphar.2018.12.014 30529194

[B7] Aziz N. Kim M. Y. Cho J. Y. (2018). Anti-inflammatory effects of luteolin: A review of *in vitro, in vivo*, and *in silico* studies. J. Ethnopharmacol. 225, 342–358. 10.1016/j.jep.2018.05.019 29801717

[B8] Bajka B. H. Rigby N. M. Cross K. L. Macierzanka A. Mackie A. R. (2015). The influence of small intestinal mucus structure on particle transport *ex vivo* . Colloids Surf. B Biointerfaces 135, 73–80. 10.1016/j.colsurfb.2015.07.038 26241918

[B9] Batista V. L. da Silva T. F. de Jesus L. C. L. Coelho-Rocha N. D. Barroso F. A. L. Tavares L. M. (2020). Probiotics, Prebiotics, synbiotics, and paraprobiotics as a therapeutic alternative for intestinal mucositis. Front. Microbiol. 11, 544490. 10.3389/fmicb.2020.544490 33042054 PMC7527409

[B10] Boeing T. de Souza P. Speca S. Somensi L. B. Mariano L. N. B. Cury B. J. (2020). Luteolin prevents irinotecan-induced intestinal mucositis in mice through antioxidant and anti-inflammatory properties. Br. J. Pharmacol. 177 (10), 2393–2408. 10.1111/bph.14987 31976547 PMC7174882

[B11] Cheah K. Y. Howarth G. S. Bastian S. E. P. (2014a). Grape seed extract dose-responsively decreases disease severity in a rat model of mucositis; Concomitantly enhancing chemotherapeutic effectiveness in colon cancer cells. PloS One 9 (1), e85184. 10.1371/journal.pone.0085184 24465501 PMC3897410

[B12] Cheah K. Y. Howarth G. S. Bindon K. A. Kennedy J. A. Bastian S. E. P. (2014b). Low molecular weight procyanidins from grape seeds enhance the impact of 5-fluorouracil chemotherapy on caco-2 human colon cancer cells. PloS One 9 (6), e98921. 10.1371/journal.pone.0098921 24905821 PMC4048230

[B13] Chen H. T. Zhang F. Li R. R. Liu Y. Wang X. Y. Zhang X. J. (2020a). Berberine regulates fecal metabolites to ameliorate 5-fluorouracil induced intestinal mucositis through modulating gut microbiota. Biomed. Pharmacother. 124, 109829. 10.1016/j.biopha.2020.109829 31958765

[B14] Chen K. J. Huang Y. L. Kuo L. M. Chen Y. T. Hung C. F. Hsieh P. W. (2022). Protective role of casuarinin from *Melastoma malabathricum* against a mouse model of 5-fluorouracil-induced intestinal mucositis: Impact on inflammation and gut microbiota dysbiosis. Phytomedicine. 101, 154092. 10.1016/j.phymed.2022.154092 35430483

[B15] Chen K. Yang R. Shen F. Q. Zhu H. L. (2020b). Advances in pharmacological activities and mechanisms of glycyrrhizic acid. Curr. Med. Chem. 27 (36), 6219–6243. 10.2174/0929867325666191011115407 31612817

[B16] Chen X. P. Yu J. Zhong B. L. Lu J. H. Lu J. J. Li S. J. (2019). Pharmacological activities of dihydrotanshinone I, a natural product from *Salvia miltiorrhiza* Bunge. Pharmacol. Res. 145, 104254. 10.1016/j.phrs.2019.104254 31054311

[B17] Chen Y. L. Zheng H. Zhang J. Z. Wang L. Jin Z. X. Gao W. Y. (2016). Reparative activity of costunolide and dehydrocostus in a mouse model of 5-fluorouracil induced intestinal mucositis. RSC Adv. 6 (7), 5249–5258. 10.1039/c5ra22371g

[B18] da Silva K. S. da Silveira B. C. Bueno L. R. da Silva L. C. M. Fonseca L. D. Fernandes E. S. (2021). Beneficial effects of polysaccharides on the epithelial barrier function in intestinal mucositis. Front. Physiol. 12, 714846. 10.3389/fphys.2021.714846 34366901 PMC8339576

[B19] Dahlgren D. Sjoblom M. Hellstrom P. M. Lennernas H. (2021). Chemotherapeutics-induced intestinal mucositis: Pathophysiology and potential treatment strategies. Front. Pharmacol. 12, 681417. 10.3389/fphar.2021.681417 34017262 PMC8129190

[B20] de Freitas-Blanco V. S. Monteiro K. M. de Oliveira P. R. de Oliveira E. C. S. Braga L. E. D. de Carvalho J. E. (2019). Spilanthol, the principal alkylamide from *Acmella oleracea*, attenuates 5-fluorouracil-induced intestinal mucositis in mice. Planta Med. 85 (3), 203–209. 10.1055/a-0715-2002 30153691

[B21] Dinda B. Dinda S. DasSharma S. Banik R. Chakraborty A. Dinda M. (2017). Therapeutic potentials of baicalin and its aglycone, baicalein against inflammatory disorders. Eur. J. Med. Chem. 131, 68–80. 10.1016/j.ejmech.2017.03.004 28288320

[B22] Escobar A. Perez M. Romanelli G. Blustein G. (2020). Thymol bioactivity: A review focusing on practical applications. Arab. J. Chem. 13 (12), 9243–9269. 10.1016/j.arabjc.2020.11.009

[B23] Feng G. Sun B. Li T. Z. (2015). Daidzein attenuates lipopolysaccharide-induced acute lung injury via toll-like receptor 4/NF-kappaB pathway. Int. Immunopharmacol. 26 (2), 392–400. 10.1016/j.intimp.2015.04.002 25887269

[B24] Fideles L. D. de Miranda J. A. L. Martins C. D. Barbosa M. L. L. Pimenta H. B. Pimentel P. V. D. (2020). Role of rutin in 5-fluorouracil-induced intestinal mucositis: Prevention of histological damage and reduction of inflammation and oxidative stress. Molecules 25 (12), 2786. 10.3390/molecules25122786 PMC7356626 32560278

[B25] Fu Y. H. Hu X. Y. Cao Y. G. Zhang Z. C. Zhang N. S. (2015). Saikosaponin A inhibits lipopolysaccharide-oxidative stress and inflammation in human umbilical vein endothelial cells via preventing TLR4 translocation into lipid rafts. Free Radic. Biol. Med. 89, 777–785. 10.1016/j.freeradbiomed.2015.10.407 26475038

[B26] Ganeshpurkar A. Saluja A. K. (2017). The pharmacological potential of rutin. Saudi Pharm. J. 25 (2), 149–164. 10.1016/j.jsps.2016.04.025 28344465 PMC5355559

[B27] Gong L. Wang C. Zhou H. Ma C. Li Y. Peng C. (2021). A review of pharmacological and pharmacokinetic properties of forsythiaside A. Pharmacol. Res. 169, 105690. 10.1016/j.phrs.2021.105690 34029711

[B28] Hewlings S. J. Kalman D. S. (2017). Curcumin: A review of its effects on human health. Foods 6 (10), 92. 10.3390/foods6100092 PMC5664031 29065496

[B29] Huang X. X. Ke K. Jin W. W. Zhu Q. R. Zhu Q. C. Mei R. Y. (2022). Identification of genes related to 5-fluorouracil based chemotherapy for colorectal cancer. Front. Immunol. 13, 887048. 10.3389/fimmu.2022.887048 35784334 PMC9247273

[B30] Ikarashi N. Kon R. Sugiyama K. (2016). Aquaporins in the colon as a new therapeutic target in diarrhea and constipation. Int. J. Mol. Sci. 17 (7), 1172. 10.3390/ijms17071172 PMC4964543 27447626

[B31] Jin S. Z. Guan T. X. Wang S. Hu M. X. Liu X. Y. Huang S. Q. (2022). Dioscin alleviates cisplatin-induced mucositis in rats by modulating gut microbiota, enhancing intestinal barrier function and attenuating TLR4/NF-kappa B signaling cascade. Int. J. Mol. Sci. 23 (8), 4431. 10.3390/ijms23084431 35457248 PMC9025408

[B32] Kawashima R. Kawakami F. Maekawa T. Yamamoto H. Koizumi W. Ichikawa T. (2015). Elemental diet moderates 5-fluorouracil-induced gastrointestinal mucositis through mucus barrier alteration. Cancer Chemother. Pharmacol. 76 (2), 269–277. 10.1007/s00280-015-2790-z 26048344

[B33] Ke X. Hu G. H. Fang W. Y. Chen J. T. Zhang X. Yang C. B. (2015). Qing Hua Chang Yin inhibits the LPS-induced activation of the IL-6/STAT3 signaling pathway in human intestinal Caco-2 cells. Int. J. Mol. Med. 35 (4), 1133–1137. 10.3892/ijmm.2015.2083 25633437

[B34] Khokhlova E. V. Smeianov V. V. Efimov B. A. Kafarskaia L. I. Pavlova S. I. Shkoporov A. N. (2012). Anti-inflammatory properties of intestinal bifidobacterium strains isolated from healthy infants. Microbiol. Immunol. 56 (1), 27–39. 10.1111/j.1348-0421.2011.00398.x 22040047

[B35] Kishore V. Yarla N. S. Bishayee A. Putta S. Malla R. Neelapu N. R. R. (2017). Multi-targeting andrographolide and its natural analogs as potential therapeutic agents. Curr. Top. Med. Chem. 17 (8), 845–857. 10.2174/1568026616666160927150452 27697058

[B36] Kissow H. (2015). Glucagon-like peptides 1 and 2: Intestinal hormones implicated in the pathophysiology of mucositis. Curr. Opin. Support. Palliat. Care 9 (2), 196–202. 10.1097/SPC.0000000000000132 25872118

[B37] Kumar A. EkavaliChopra K. Mukherjee M. Pottabathini R. Dhull D. K. (2015). Current knowledge and pharmacological profile of berberine: An update. Eur. J. Pharmacol. 761, 288–297. 10.1016/j.ejphar.2015.05.068 26092760

[B38] Lang W. Y. Cheng M. Zheng X. Zhao Y. P. Qu Y. L. Jia Z. (2022). Forsythiaside A alleviates methotrexate-induced intestinal mucositis in rats by modulating the NLRP3 signaling pathways. Int. Immunopharmacol. 103, 108466. 10.1016/j.intimp.2021.108466 34933162

[B39] Lee H. S. Lee J. Smolensky D. Lee S. H. (2020). Potential benefits of patchouli alcohol in prevention of human diseases: A mechanistic review. Int. Immunopharmacol. 89 (A), 107056. 10.1016/j.intimp.2020.107056 33039955 PMC7543893

[B40] Lei W. R. Liu D. X. Sun M. Lu C. X. Yang W. W. Wang C. Y. (2021). Targeting STAT3: A crucial modulator of sepsis. J. Cell. Physiol. 236 (11), 7814–7831. 10.1002/jcp.30394 33885157

[B41] Li C. L. Ai G. X. Wang Y. F. Lu Q. Luo C. D. Tan L. H. (2020). Oxyberberine, a novel gut microbiota-mediated metabolite of berberine, possesses superior anti-colitis effect: Impact on intestinal epithelial barrier, gut microbiota profile and TLR4-MyD88-NF-kappa B pathway. Pharmacol. Res. 152, 104603. 10.1016/j.phrs.2019.104603 31863867

[B42] Li H. L. Lu L. Wang X. S. Qin L. Y. Wang P. Qiu S. P. (2017). Alteration of gut microbiota and inflammatory cytokine/chemokine profiles in 5-fluorouracil induced intestinal mucositis. Front. Cell. Infect. Microbiol. 7, 455. 10.3389/fcimb.2017.00455 29124041 PMC5662589

[B43] Li H. Y. Gao C. D. Liu C. Liu L. J. Zhuang J. Yang J. (2021). A review of the biological activity and pharmacology of cryptotanshinone, an important active constituent in Danshen. Biomed. Pharmacother. 137, 111332. 10.1016/j.biopha.2021.111332 33548911

[B44] Li Y. Yao J. Y. Han C. Y. Yang J. X. Chaudhry M. T. Wang S. N. (2016). Quercetin, inflammation and immunity. Nutrients 8 (3), 167. 10.3390/nu8030167 26999194 PMC4808895

[B45] Lin N. Xu L. F. Sun M. (2013). The protective effect of trefoil factor 3 on the intestinal tight junction barrier is mediated by toll-like receptor 2 *via* a pi3k/akt dependent mechanism. Biochem. Biophys. Res. Commun. 440 (1), 143–149. 10.1016/j.bbrc.2013.09.049 24051092

[B46] Liu X. N. Li H. M. Wang S. P. Zhang J. Z. Liu D. L. (2021). Sesquiterpene lactones of *Aucklandia lappa*: Pharmacology, pharmacokinetics, toxicity, and structure-activity relationship. Chin. Herb. Med. 13 (2), 167–176. 10.1016/j.chmed.2020.11.005 36117502 PMC9476744

[B47] Lotfi M. Kazemi S. Shirafkan F. Hosseinzadeh R. Ebrahimpour A. Barary M. (2021). The protective effects of quercetin nano-emulsion on intestinal mucositis induced by 5-fluorouracil in mice. Biochem. Biophys. Res. Commun. 585, 75–81. 10.1016/j.bbrc.2021.11.005 34800883

[B48] Luqman S. Pezzuto J. M. (2010). NFkappaB: A promising target for natural products in cancer chemoprevention. Phytother. Res. 24 (7), 949–963. 10.1002/ptr.3171 20577970

[B49] Mi H. Dong Y. Zhang B. Wang H. N. Peter C. C. K. Gao P. (2017). Bifidobacterium infantis ameliorates chemotherapy-induced intestinal mucositis via regulating T cell immunity in colorectal cancer rats. Cell. Physiol. biochem. 42 (6), 2330–2341. 10.1159/000480005 28848081

[B50] Miknevicius P. Zulpaite R. Leber B. Strupas K. Stiegler P. Schemmer P. (2021). The impact of probiotics on intestinal mucositis during chemotherapy for colorectal cancer: A comprehensive review of animal studies. Int. J. Mol. Sci. 22 (17), 9347. 10.3390/ijms22179347 34502251 PMC8430988

[B51] Panwar S. Sharma S. Tripathi P. (2021). Role of barrier integrity and dysfunctions in maintaining the healthy gut and their health outcomes. Front. Physiol. 12, 715611. 10.3389/fphys.2021.715611 34630140 PMC8497706

[B52] Prisciandaro L. D. Geier M. S. Butler R. N. Cummins A. G. Howarth G. S. (2011). Evidence supporting the use of probiotics for the prevention and treatment of chemotherapy-induced intestinal mucositis. Crit. Rev. Food Sci. Nutr. 51 (3), 239–247. 10.1080/10408390903551747 21390944

[B53] Reinke D. Kritas S. Polychronopoulos P. Skaltsounis A. L. Aligiannis N. Tran C. D. (2015). Herbal substance, acteoside, alleviates intestinal mucositis in mice. Gastroenterol. Res. Pract. 2015, 327872. 10.1155/2015/327872 25628651 PMC4300033

[B54] Ribeiro R. A. Wanderley C. W. S. Wong D. V. T. Mota J. M. S. C. Leite C. A. V. G. Souza M. H. L. P. (2016). Irinotecan- and 5-fluorouracil-induced intestinal mucositis: Insights into pathogenesis and therapeutic perspectives. Cancer Chemother. Pharmacol. 78 (5), 881–893. 10.1007/s00280-016-3139-y 27590709

[B55] Sharifi-Rad M. Varoni E. M. Iriti M. Martorell M. Setzer W. N. Contreras M. D. (2018). Carvacrol and human health: A comprehensive review. Phytother. Res. 32 (9), 1675–1687. 10.1002/ptr.6103 29744941

[B56] Silveira N. Sandjo L. P. Biavatti M. W. (2018). Spilanthol-containing products: A patent review (1996-2016). Trends Food Sci. Technol. 74, 107–111. 10.1016/j.tifs.2018.02.012

[B57] Skamrahl M. Pang H. T. Ferle M. Gottwald J. Rubeling A. Maraspini R. (2021). Tight junction ZO proteins maintain tissue fluidity, ensuring efficient collective cell migration. Adv. Sci. 8 (19), 2100478. 10.1002/advs.202100478 PMC8498871 34382375

[B58] Sougiannis A. T. VanderVeen B. N. Davis J. M. Fan D. P. Murphy E. A. (2021). Understanding chemotherapy-induced intestinal mucositis and strategies to improve gut resilience. Am. J. Physiol. Gastrointest. Liver Physiol. 320 (5), G712–G719. 10.1152/ajpgi.00380.2020 33471628 PMC8202195

[B59] Sukhotnik I. Moati D. Shaoul R. Loberman B. Pollak Y. Schwartz B. (2018). Quercetin prevents small intestinal damage and enhances intestinal recovery during methotrexate-induced intestinal mucositis of rats. Food Nutr. Res. 62, 1327. 10.29219/fnr.v62.1327 PMC5883860 30026677

[B60] Swamy M. K. Sinniah U. R. (2015). A comprehensive review on the phytochemical constituents and pharmacological activities of *Pogostemon cablin* Benth.: An aromatic medicinal plant of industrial importance. Molecules 20 (5), 8521–8547. 10.3390/molecules20058521 25985355 PMC6272783

[B61] Takeuchi O. Akira S. (2002). MyD88 as a bottle neck in Toll/IL-1 signaling. Curr. Top. Microbiol. Immunol. 270, 155–167. 10.1007/978-3-642-59430-4_10 12467250

[B62] Tanaka H. Takechi M. Kiyonari H. Shioi G. Tamura A. Tsukita S. (2015). Intestinal deletion of Claudin-7 enhances paracellular organic solute flux and initiates colonic inflammation in mice. Gut 64 (10), 1529–1538. 10.1136/gutjnl-2014-308419 25691495

[B63] Tappenden K. A. (2008). Inflammation and intestinal function: Where does it start and what does it mean? JPEN. J. Parenter. Enter. Nutr. 32 (6), 648–650. 10.1177/0148607108325177 18974246

[B64] Thorpe D. Butler R. Sultani M. Vanhoecke B. Stringer A. (2020). Irinotecan-induced mucositis is associated with goblet cell dysregulation and neural cell damage in a tumour bearing DA rat model. Pathol. Oncol. Res. 26 (2), 955–965. 10.1007/s12253-019-00644-x 30919275

[B65] Tooley K. L. Howarth G. S. Butler R. N. (2009). Mucositis and non-invasive markers of small intestinal function. Cancer Biol. Ther. 8 (9), 753–758. 10.4161/cbt.8.9.8232 19276675

[B66] van Vliet M. J. Harmsen H. J. M. de Bont E. S. J. M. Tissing W. J. E. (2010). The role of intestinal microbiota in the development and severity of chemotherapy-induced mucositis. PLoS Pathog. 6 (5), e1000879. 10.1371/journal.ppat.1000879 20523891 PMC2877735

[B67] Varankovich N. V. Nickerson M. T. Korber D. R. (2015). Probiotic-based strategies for therapeutic and prophylactic use against multiple gastrointestinal diseases. Front. Microbiol. 6, 685. 10.3389/fmicb.2015.00685 26236287 PMC4500982

[B68] Wang J. Zhao H. Lv K. Zhao W. Zhang N. Yang F. (2021a). Pterostilbene ameliorates DSS-induced intestinal epithelial barrier loss in mice *via* suppression of the NF-kappa B-mediated MLCK-MLC signaling pathway. J. Agric. Food Chem. 69 (13), 3871–3878. 10.1021/acs.jafc.1c00274 33759516

[B69] Wang L. Song B. H. Hu Y. Chen J. Zhang S. S. Chen D. P. (2021b). Puerarin ameliorates 5-fluorouracil-induced intestinal mucositis in mice by inhibiting JAKs. J. Pharmacol. Exp. Ther. 379 (2), 147–155. 10.1124/jpet.121.000677 34400527

[B70] Wang L. Wang R. Wei G. Y. Wang S. M. Du G. H. (2020a). Dihydrotanshinone attenuates chemotherapy-induced intestinal mucositis and alters fecal microbiota in mice. Biomed. Pharmacother. 128, 110262. 10.1016/j.biopha.2020.110262 32447214

[B71] Wang L. Wang R. Wei G. Y. Wang S. M. Du G. H. (2020b2020). Study on the therapeutic effects and mechanism of cryptotanshinone on mice with chemotherapy-induced mucositis. Acta Pharm. Sin. 55 (8), 1801–1811.

[B72] Wang R. Wang L. Wei G. Y. Liu N. N. Zhang L. Wang S. M. (2020c2020). The effect and mechanism of baicalein on regulating gut microbiota and improving chemotherapy-induced intestinal mucositis in mice. Acta Pharm. Sin. 55 (5), 868–876.

[B73] Wang X. Y. Zhang B. Lu Y. Xu L. Wang Y. J. Cai B. Y. (2021). RNA-seq and *in vitro* experiments reveal the protective effect of curcumin against 5-fluorouracil-induced intestinal mucositis *via* IL-6/STAT3 signaling pathway. J. Immunol. Res. 2021, 8286189. 10.1155/2021/8286189 34337082 PMC8318760

[B74] Wang Y. Y. Wei B. Wang D. P. Wu J. J. Gao J. H. Zhong H. Q. (2022). DNA damage repair promotion in colonic epithelial cells by andrographolide downregulated cGAS-STING pathway activation and contributed to the relief of CPT-11-induced intestinal mucositis. Acta Pharm. Sin. B 12 (1), 262–273. 10.1016/j.apsb.2021.03.043 35127384 PMC8799857

[B75] Wright T. H. Yazbeck R. Lymn K. A. Whitford E. J. Cheah K. Y. Butler R. N. (2009). The herbal extract, Iberogast, improves jejunal integrity in rats with 5-Fluorouracil (5-FU)-induced mucositis. Cancer Biol. Ther. 8 (10), 923–929. 10.4161/cbt.8.10.8146 19276679

[B76] Wu J. Z. Gan Y. X. Li M. X. Chen L. P. Liang J. L. Zhuo J. Y. (2020). Patchouli alcohol attenuates 5-fluorouracil-induced intestinal mucositis *via* TLR2/MyD88/NF-kB pathway and regulation of microbiota. Biomed. Pharmacother. 124, 109883. 10.1016/j.biopha.2020.109883 32004938

[B77] Wu J. Z. Gan Y. X. Luo H. J. Xu N. Chen L. P. Li M. Y. (2021). β-Patchoulene ameliorates water transport and the mucus barrier in 5-fluorouracil-induced intestinal mucositis rats *via* the cAMP/PKA/CREB signaling pathway. Front. Pharmacol. 12, 689491. 10.3389/fphar.2021.689491 34512326 PMC8424048

[B78] Wu Y. P. Zhu C. Chen Z. Chen Z. J. Zhang W. N. Ma X. Y. (2016). Protective effects of *Lactobacillus plantarum* on epithelial barrier disruption caused by enterotoxigenic *Escherichia coli* in intestinal porcine epithelial cells. Vet. Immunol. Immunopathol. 172, 55–63. 10.1016/j.vetimm.2016.03.005 27032504

[B79] Xiang D. C. Yang J. Y. Xu Y. J. Zhang S. Li M. Zhu C. (2020). Protective effect of andrographolide on 5-Fu induced intestinal mucositis by regulating p38 MAPK signaling pathway. Life Sci. 252, 117612. 10.1016/j.lfs.2020.117612 32247004

[B80] Xiong Y. J. Deng Z. B. Liu J. N. Qiu J. J. Guo L. Feng P. P. (2019). Enhancement of epithelial cell autophagy induced by sinensetin alleviates epithelial barrier dysfunction in colitis. Pharmacol. Res. 148, 104461. 10.1016/j.phrs.2019.104461 31542404

[B81] Yeung C. Y. Chiau J. S. C. Cheng M. L. Chan W. T. Chang S. W. Chang Y. H. (2020). Modulations of probiotics on gut microbiota in a 5-fluorouracil-induced mouse model of mucositis. J. Gastroenterol. Hepatol. 35 (5), 806–814. 10.1111/jgh.14890 31674687

[B82] Yoneda J. Nishikawa S. Kurihara S. (2021). Oral administration of cystine and theanine attenuates 5-fluorouracil-induced intestinal mucositis and diarrhea by suppressing both glutathione level decrease and ROS production in the small intestine of mucositis mouse model. BMC Cancer 21 (1), 1343. 10.1186/s12885-021-09057-z 34922485 PMC8684148

[B83] Yue B. Gao R. Y. Lv C. Yu Z. L. Wang H. Geng X. L. (2021). Berberine improves irinotecan-induced intestinal mucositis without impairing the anti-colorectal cancer efficacy of irinotecan by inhibiting bacterial beta-glucuronidase. Front. Pharmacol. 12, 774560. 10.3389/fphar.2021.774560 34795594 PMC8593678

[B84] Zeeshan M. Atiq A. Ul Ain Q. Ali J. Khan S. Ali H. (2021). Evaluating the mucoprotective effects of glycyrrhizic acid-loaded polymeric nanoparticles in a murine model of 5-fluorouracil-induced intestinal mucositis via suppression of inflammatory mediators and oxidative stress. Inflammopharmacology 29 (5), 1539–1553. 10.1007/s10787-021-00866-z 34420176

[B85] Zhang L. (2019). Pharmacokinetics and drug delivery systems for puerarin, a bioactive flavone from traditional Chinese medicine. Drug Deliv. 26 (1), 860–869. 10.1080/10717544.2019.1660732 31524010 PMC6758605

[B86] Zhang X. J. Yuan Z. W. Qu C. Yu X. T. Huang T. Chen P. V. (2018). Palmatine ameliorated murine colitis by suppressing tryptophan metabolism and regulating gut microbiota. Pharmacol. Res. 137, 34–46. 10.1016/j.phrs.2018.09.010 30243842

[B87] Zhang X. Wan Y. Feng J. Li M. Jiang Z. (2021). Involvement of TLR2/4-MyD88-NF-kappa B signaling pathway in the pathogenesis of intracranial aneurysm. Mol. Med. Rep. 23 (4), 230. 10.3892/mmr.2021.11869 33655339

[B88] Zhao Q. Maynard C. L. (2022). Mucus, commensals, and the immune system. Gut Microbes 14 (1), 2041342. 10.1080/19490976.2022.2041342 35239459 PMC8903774

[B89] Zhou Y. Q. Zhu J. L. Shao L. Y. Guo M. M. (2020). Current advances in acteoside biosynthesis pathway elucidation and biosynthesis. Fitoterapia 142, 104495. 10.1016/j.fitote.2020.104495 32045692

